# Room Temperature Defluorination of Poly(tetrafluoroethylene) by a Magnesium Reagent

**DOI:** 10.1021/jacs.3c02526

**Published:** 2023-05-08

**Authors:** Daniel J. Sheldon, Joseph M. Parr, Mark R. Crimmin

**Affiliations:** Department of Chemistry, Molecular Sciences Research Hub, Imperial College London, 82 Wood Lane, Shepherds Bush, London, W12 0BZ, U.K.

## Abstract

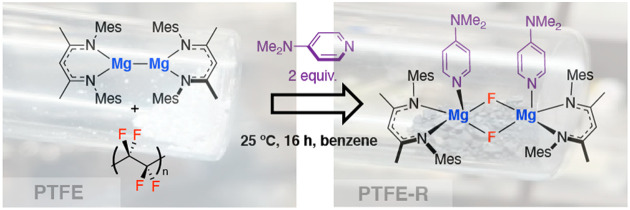

Perfluoroalkyl substances (PFAS) are pervasive in the environment. The largest single use material within the PFAS compound class is poly(tetrafluoroethylene) (PTFE), a robust and chemically resistant polymer. Despite their widespread use and serious concerns about their role as pollutants, methods for repurposing PFAS are rare. Here we show that a nucleophilic magnesium reagent reacts with PTFE at room temperature, generating a molecular magnesium fluoride which is easily separated from the surface-modified polymer. The fluoride in turn can be used to transfer the fluorine atoms to a small array of compounds. This proof-of-concept study demonstrates that the atomic fluorine content of PTFE can be harvested and reused in chemical synthesis.

Poly(tetrafluoro)ethylene (PTFE) is produced on an estimated 2 × 10^8^ kg/year scale through a multistep process that converts fluorite (CaF_2_) to HF to CHClF_2_ to C_2_F_4_, which is then subject to a radical polymerization.^1^ PTFE is a high-molecular weight semicrystalline polymer and is among the most chemically resistant materials in existence. PTFE finds many applications such as a wire coating and insulator, in nonstick cookware and in weather-resistant materials. Despite recent advances in destruction methods for per- and polyfluoroalkyl substances (PFAS),^2^ current approaches for recycling or destruction of PTFE are limited and there is an urgent need to establish methods for its remediation.

Depolymerization of PTFE to C_2_F_4_ (and related low molecular weight fluorocarbons) by vacuum pyrolysis is an energy intensive process; at 510 °C the half-life of conversion of PTFE to monomers has been estimated as ∼30 min.^3^ Although this provides a viable route for reprocessing of PTFE, it is not an effective approach to recycle this material in any other way. Defluorination methods could harvest fluorine atoms from PTFE while also creating structurally modified materials. This approach could allow waste PTFE to be directly used as a fluorine source in chemical synthesis, providing a more sustainable alternative to current synthetic methods that almost exclusively rely on hazardous HF, derived from mineral sources of CaF_2_. Chemical methods for defluorinating PTFE include reactions with Mg in supercritical CO_2_ above 510 °C,^4^ Ca(OH)_2_ in supercritical water above 650 °C,^5^ with zinc powder as a template at 700 °C,^6^ or with Mg_2_Si at 600 °C to form silicon carbide nanoparticles.^7^ Low temperature reactions (<100 °C) require strong reductants such as s-block metals in liquid ammonia, lithium–mercury amalgam, sodium naphthalide, benzoin dianion, or alkyl lithium reagents.^8−17^ At the most extreme, destruction of PTFE occurs in military grade pyrotechnic materials such as Magnesium-Teflon-Viton pyrolants.^18^ Inorganic metal fluoride MF_(s)_ (M = Li, Na or K) or MF_2(s)_ (M = Mg, Ca) is often proposed as a reaction byproduct, but only in a limited number of cases have these compounds been confirmed by spectroscopic methods (e.g., powder XRD, XPS, SS ^7^Li NMR).

Here we report the controlled defluorination of PTFE powder with a molecular magnesium-based reducing agent under extremely mild conditions (25 °C, 1 atm). The approach allows the formation of a hydrocarbon-soluble magnesium fluoride coordination compound which is easily separable and recoverable from the modified PTFE. This kinetically stabilized species can be used as a fluoride carrier and is expected to be more reactive than inorganic metal fluorides, MF_(s)_ or MF_2(s)_, which suffer from high lattice enthalpies and high stability. We show that PTFE derived molecular fluoride can be used as a fluorinating agent, establishing proof-of-concept for the direct reuse of the atomic fluorine content of PTFE in synthesis (Figure 1).

**Figure 1:**
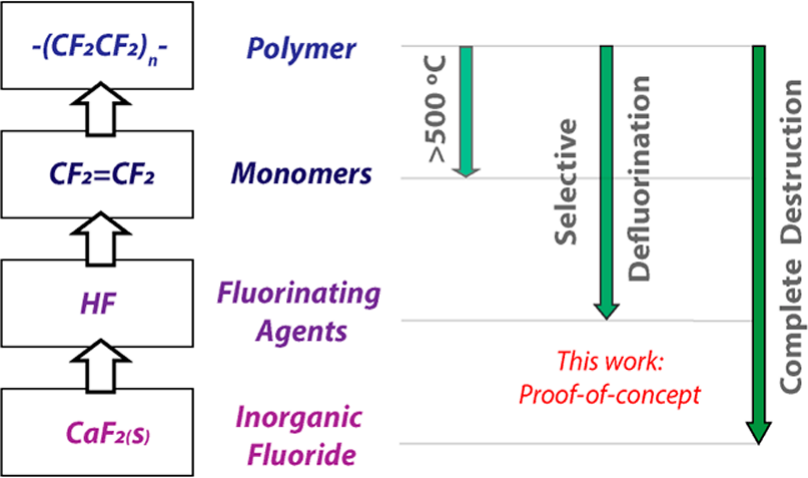
Product-chain for production of PTFE, along with potential routes for recycling including: thermal depolymerization (above 500 °C), selective defluorination (this work) to form fluorinating agents, and complete destruction back to inorganic metal fluoride.

**PTFE** powder (250 mg, 1 μm particle size, Sigma-Aldrich, *M*_*n*_ = 10^6^–10^7^, 8 equiv of repeat units), **1** (250 mg, 0.35 mmol), 4-(dimethylamino)pyridine (DMAP 85 mg, 0.70 mmol), and benzene (10 mL) were combined under an inert atmosphere of argon. Jones and co-workers have shown that the magnesium nucleophile used in this reaction, **1**,^19,20^ displays enhanced reactivity on the addition of a Lewis base.^21,22^ The reaction mixture was agitated and left to proceed for 16 h at 25 °C. The red solution turns dark red-brown as the reaction progresses, PTFE remains undissolved throughout, and it is assumed the process is heterogeneous. The reaction mixture was filtered to remove PTFE and the mother liquor was concentrated to a solid, which was identified by multinuclear NMR spectroscopy to be **2** (Figure 2). Recrystallization of the crude product was achieved by slow evaporation of a concentrated benzene solution. **2** was isolated in 85% yield based on **1** as the limiting agent. **2** demonstrates a characteristic resonance at δ = −183.9 ppm in the ^19^F NMR spectrum. In the solid-state, **2** demonstrates a dimeric structure in which a pair of five-coordinate magnesium sites supported by terminal β-diketiminate and DMAP ligands are bridged by fluoride atoms derived from **PTFE** (Figure 2).^23^

**Figure 2:**
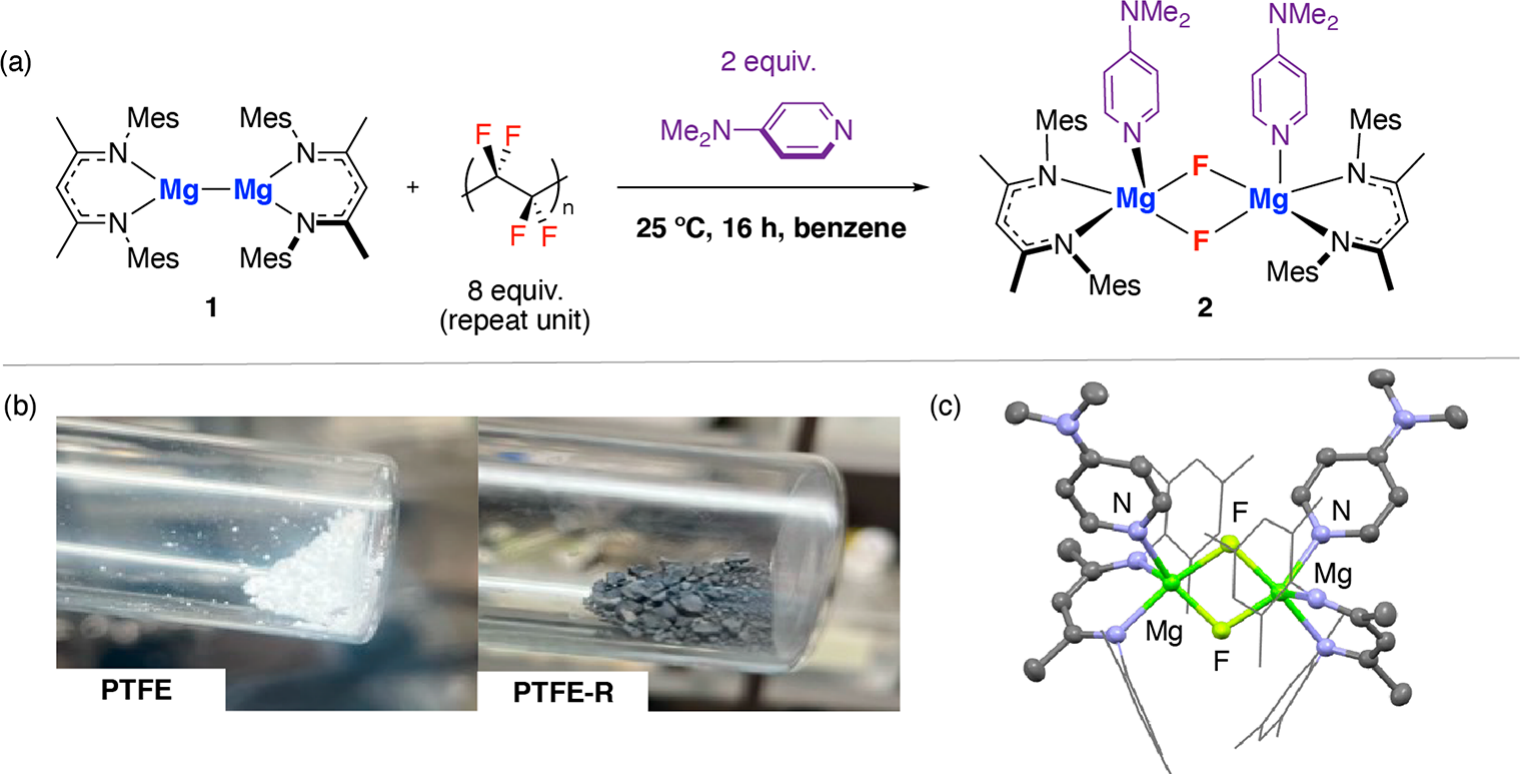
(a) Room temperature reaction of **1** with PTFE. (b) Samples of PTFE and PTFE-R before and after reaction, respectively. (c) Single-crystal X-ray structure of **2** isolated from PTFE.

The tolerance of the reaction with **1** on the source and any pretreatment of **PTFE** was investigated. **PTFE** (1 μm) from different commercial suppliers (Sigma-Aldrich, Alfa Aesar), with or without a Soxhlet extraction (toluene or THF) prior to reduction behaved similarly, reacting with **1** in >70% yield.^24^ Modification of the particle size, however, impacted the efficiency of the reaction. A series of particle sizes were investigated (1 μm, 6–9 μm, 15–25 μm, 35 and 200 μm), with larger particle sizes of **PTFE** generally leading to lower and longer reaction times. **2** could also be obtained from reaction of **1** + DMAP with poly(vinylidenedifluoride) (PVDF), albeit in a lower yield of 40%, determined after exhaustion of **1**.

Following the reaction with **1** + DMAP, **PTFE** is converted from a colorless solid to a gray powder which we label **PTFE-R**. The polymer remained and beyond **2** no further fluorinated products are observed in solution by ^19^F NMR spectroscopy. Solid-state magic angle spinning (SS-MAS) NMR spectroscopic data were collected on **PTFE** and **PTFE-R**, and both samples show characteristic resonances at δ = −124 ppm and δ = 110 ppm in the ^19^F and ^13^C NMR spectrum respectively consistent with little change to the bulk sample on reaction with **1** (Figure 3a). Powder X-ray diffraction (XRD) of **PTFE** shows a reflection at 2θ = 18° assigned to a crystalline phase along with a diffuse scattering peak 2θ = 40°, which has been assigned as an amorphous component. **PTFE-R** retains the reflection at 2θ = 18°; however, the intensity of the 2θ = 40° peak is greatly reduced. Scanning electron microscopy (SEM) of **PTFE** and **PTFE-R** show changes to the surface of the polymer following reaction with **1** (Figure 3b). In combination, the SS-MAS NMR, powder XRD, and SEM data suggest that the most likely reactive sites of **PTFE** are in amorphous regions on the polymer surface.

**Figure 3:**
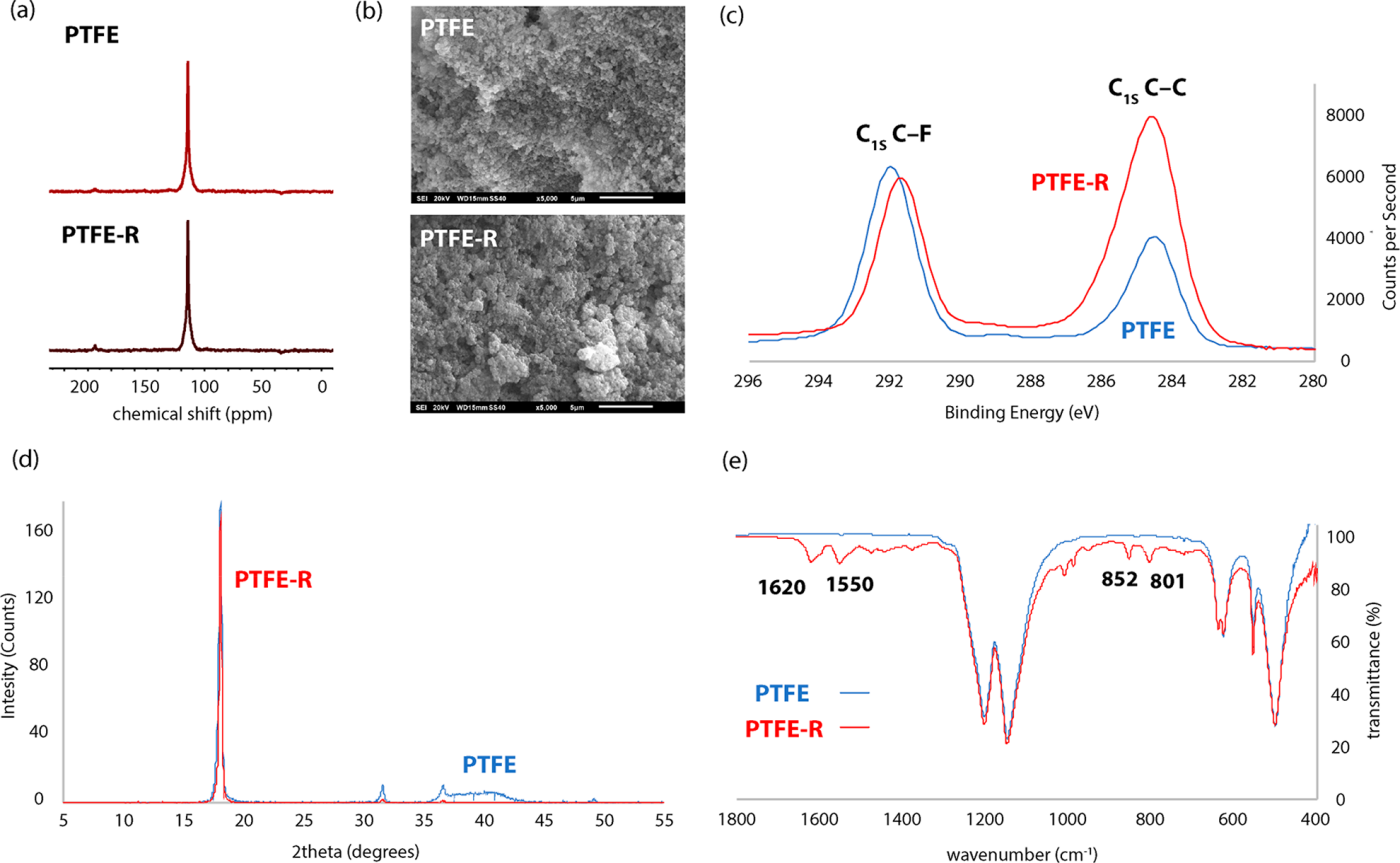
Solid-state data on **PTFE** and **PTFE-R**. (a) ^13^C SS-MAS NMR data, (b) SEM images, (c) XPS data, (d) powder XRD, and (e) ATR-IR spectroscopic data.

Surface sensitive techniques were used to further interrogate the changes to **PTFE** on reaction with **1**. X-ray Photoelectron Spectroscopy (XPS) measurements show the expected transitions characteristic of −CF_2_– functional groups from the F(1S) orbitals (689.8 eV) and C(1S) orbitals (292.0 and 284.4 eV) of **PTFE** (Figure 3c).^25,26^ The same transitions are also observed in **PTFE-R**; however, integration of peaks in the C(1s) scan shows that the C–F:C–C ratio has changed from 1:0.5 to 1:1.4 consistent with lower fluorine content near the surface of **PTFE-R** compared to **PTFE**. In addition, a new transition is observed at 684.7 eV assigned to a Mg bound F(1S) environment (Supporting Information, Figure S9). Hence, while it is clear from the high recovery of **2** that most of the exercised fluorine content is transferred to the solution phase, we cannot discount the retention of small amounts of metal fluoride species at the surface of **PTFE-R**.

Attenuated total reflectance IR spectroscopy also shows clear differences between **PTFE** and **PTFE-R**. **PTFE-R** shows vibrations 1620, 1550, 1006, 985, 852, and 801 cm^–1^ not present in **PTFE**, which most likely derive from C=C stretching and bending modes of newly created alkene functional groups (Figure 3e).^27^ To support this assignment, a sample of **PTFE-R** was reacted with an excess of BH_3_·THF in THF for 72 h. Following reisolation of the polymer, the key vibrations assigned to C=C modes are no longer present (Supporting Information, Figure S7). The XPS and IR data suggest that defluorination occurs at the surface of PTFE, most likely with the creation of alkene functionality through a 1,2-defluorination of polymer chains.

To further probe the potential formation of unsaturated C=C bonds through 1,2-defluorination, **1** was reacted with perfluoro(methylcyclohexane), a short-chain PFAS, in the presence of DMAP. After 3 h at 25 °C, **2** was formed (90% yield by NMR spectroscopy) alongside **3** (10% yield by NMR spectroscopy), giving clear evidence for the formation of unsaturated C=C bonds (Figure 4). Similar defluorination reactions with perfluoroalkanes are described in the literature.^28−30^ Despite being formed in small amounts, observation of **3** is explained from a stepwise defluorination of perfluoro(methylcyclohexane) to form octafluorotoluene, which is proposed to react further with **1** to effect metalation at the 4-position of the newly unsaturated ring system, allowing trapping of the organic fragment to form **3** (Figure 4). A control reaction between octafluorotoluene, **1**, and DMAP also formed **3**.

**Figure 4:**
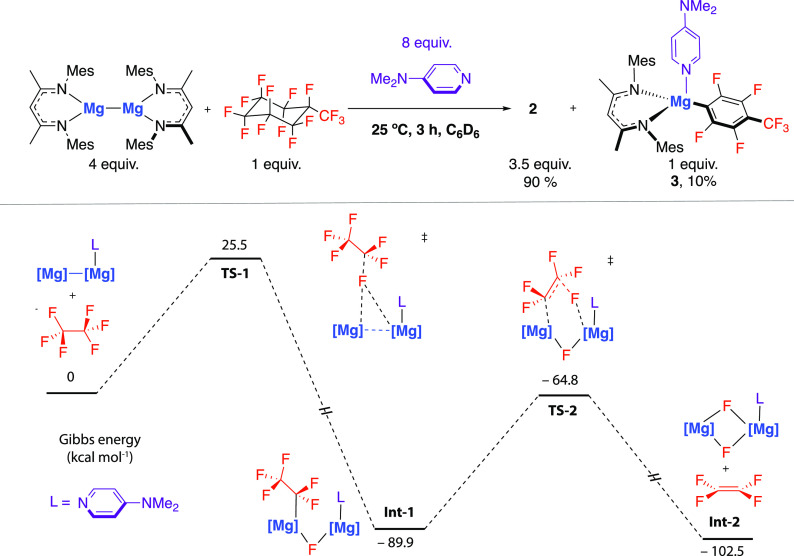
(a) Reaction of **1** with perfluoromethylcyclohexane. (b) DFT calculated mechanism for the 1,2-defluorination of PFAS with **1**.

DFT calculations were conducted to establish a feasible mechanistic model for the 1,2-defluorination of PFAS using **1** + DMAP. C_2_F_6_ was used as a model for PTFE and perfluoro(methylcyclohexane), where both site selectivity (1° vs 2°) and conformational flexibility are complicating factors. Binding of DMAP to **1** was assumed to be fast and reversible under the reaction conditions. Fluxionality in ^1^H NMR spectroscopic data for **1·DMAP** suggests the DMAP can move rapidly between Mg centers.^21,22^ Both **1·DMAP** and **1·DMAP**_**2**_ could be prepared from **1** and DMAP,^31^ and their structures were confirmed by single crystal X-ray diffraction (Supporting Information, Figure S2). The calculations suggest an initial defluorination of C_2_F_6_ by **1** + DMAP occurs by attack of **1·DMAP** at the fluorine atom of C_2_F_6_ via **TS-1** (Δ*G*^‡^_298 K_ = 25.5 kcal mol^–1^) to form **Int-1**. This mode of reactivity has been established for **1** in the absence of DMAP but appears to be accelerated by the presence of this additive.^32^ The transition state for C–F bond breaking is asymmetric and the three-coordinate Mg center of **1·DMAP** acts as a nucleophilic site, while the 4-coordinate Mg center acts as a fluoride acceptor. **Int-1** can undergo a second fluoride elimination via **TS-2** (Δ*G*^‡^_298 K_ = 25.1 kcal mol^–1^) to create a C=C bond and form C_2_F_4_, and reaction of the magnesium fluoride byproduct with a second equivalent of DMAP forms **2**. The overall reaction is highly exergonic (Δ*G*°_298 K_ = −102.5 kcal mol^–1^). **TS-1** was further analyzed by natural bond orbital (NBO), revealing charge accumulation on the C and F atoms of the breaking C–F bond as the transition state is approached, while positive charge accumulates at the three-coordinate Mg atom. The analysis is consistent with a flow of electrons from the main group reagent to C_2_F_6_.

The reaction of C_2_F_6_ with either **1** (Δ*G*^‡^_298 K_ = 30.2 kcal mol^–1^) or **1·(DMAP)**_**2**_ (Δ*G*^‡^_298 K_ = 38.2 kcal mol^–1^) was calculated to proceed with a higher activation energy barrier than **1·DMAP** (Supporting Information, Figure S16). It is likely that polarization and stretching of the Mg–Mg bond in **1·DMAP** compared to the symmetric species leads to enhanced reactivity of this species.^22^ Lewis bases have been used in a similar way to activate diboron compounds.^33^*NPA* charges in **1·DMAP** reveal the 3-coordinate Mg atom has a more negative charge (0.84) compared to the four-coordinate Mg atom (1.07), and hence nucleophilic attack originates from the three-coordinate Mg atom. Alternative mechanisms including electron transfer were found to be less accessible by DFT calculations and are less likely to be operating under the reaction conditions (Supporting Information, Figure S18, Scheme S12).

Compound **2** is soluble in hydrocarbon solvents and is a viable fluorinating agent. Reactive fluorides have been studied extensively in nucleophilic fluorination reactions.^34−37^**2** is potentially more reactive than inorganic metal fluorides such as MgF_2(s)_ or CaF_2(s)_ as there is no need to overcome the high lattice-enthalpies for an onward reaction. **2** was used to transfer PTFE-derived fluorine atoms to a series of electrophiles. For example, **2** reacts with Me_3_SiCl to cleanly form Me_3_SiF (99%). **2** can also be used to prepare tetrafluoroborate anions (e.g., [*n*-Bu_4_N][BF_4_]) from reaction with BF_3_ and [*n*-Bu_4_NCl] (95%). While unreactive with acyl and aryl halides itself, **2** can be used to generate a molecular aluminum difluoride (99%) that has been used as a fluorinating agent to prepare benzoyl fluoride.^38^ Tetrafluoroborate anions find myriad uses in chemical synthesis, while acyl fluorides can be used to create numerous organofluorine products.^39−41^

## Supporting Information Available

Synthetic procedures, NMR data, crystallographic data, and computational methods (PDF)
Cartesian coordinates of the DFT-optimized structures (XYZ)

## Supplementary Material

ja3c02526_si_001.pdf

ja3c02526_si_002.xyz
