# Supplementary material for: Room Temperature Defluorination of Poly(tetrafluoroethylene) by a Magnesium Reagent
Source: J Am Chem Soc. 2023 May 8;145(19):10486–90. doi: 10.1021/jacs.3c02526 (PMC10197119; doi:10.1021/jacs.3c02526)
Supplement: Supplementary file 1 — ja3c02526_si_001.pdf [file ja3c02526_si_001.pdf]

Supporting information for:

Room Temperature Defluorination of Poly(tetrafluoroethylene) by a Magnesium Reagent

Daniel J. Sheldon[a], Joseph M. Parr[a], Mark R. Crimmin* [a]

*Corresponding author. Email: m.crimmin@imperial.ac.uk

[a]Molecular Sciences Research Hub, Department of Chemistry, Imperial College London, 82 Wood Lane, White City, Shepherds Bush, London, W12 0BZ, UK.

Contents

1. General Experimental 1
2. Synthetic Procedures 2
   2.1. Defluorination of **PTFE** with **1** and 4-(dimethylamino)pyridine (**DMAP**) 2
   2.2. Defluorination of **PTFE** with **1** 5
   2.3. Defluorination of poly(vinylidenedifluoride) **PVDF** with **1** and **DMAP** 6
   2.4. Defluorination of Perfluoromethylcyclohexane with **1** and **DMAP** 7
   2.5. Defluorination of other small-molecule **PFAS** with **1** and **DMAP** 9
   2.6. Isolation of **1-(DMAP)$_2$** 10
   2.7. Isolation of **1-(DMAP)** 10
3. Reactions of **2** with Electrophiles 13
4. Solid-State Characterisation of **PTFE-R** 15
5. Computational Methods 22
   5.1. Discussion of Computational Model 23
   5.2. Alternative Mechanisms 24
   5.3. NBO Data for **TS-1**, **TS-1A** and **TS-1B** 26
   5.4. Assessment of the Functional 29
   5.5. Assessment of Basis Sets 30
   5.6. XYZ Coordinates 31
6. References 41

1. General Experimental

Standard Schlenk line and glovebox techniques were used for all manipulations under an inert atmosphere of dinitrogen or argon unless otherwise stated. NMR scale reactions were performed in J. Young NMR tubes equipped with internal standard capillaries of ferrocene (^{1}H NMR spectroscopy) and prepared in a glovebox. An MBraun Labmaster glovebox was used, operating at <0.1 ppm H_2O and <0.1 ppm O_2. ^{1}H, ^{13}C, ^{19}F and ^{11}B NMR spectra were recorded on Bruker 400 MHz or 500 MHz machines, and referenced against $SiMe_4$ (^{1}H, ^{13}C), $CFCl_3$ (^{19}F) and EtO•BF_3 (^{11}B). NMR data were processed using the MestReNova software package. Infrared spectra were obtained on a Cary630 spectrometer. XPS measurements were obtained on a Thermo Scientific K-Alpha+ X-ray Photoelectron Spectrometer. SEM images were collected using a JEOL 6010LA microscope. MAS-SS-NMR measurements were carried out by Dr. Nasima Kanwal at Queen Mary University London on a Bruker 400 MHz spectrometer. Single crystal X-ray diffraction data were collected using an Agilent Xcalibur PX Ultra A diffractometer. The CrysAlisPro software was used for data collection, as well as peak hunting, indexing reflections in reciprocal space, integration of the raw frames and application of corrections including interframe scaling, Lorentz, flood field and dark current corrections. The structures were solved using the SHELXT program and least-square refined using the SHELXL program within the Olex2 system suite.[1,2] Solvents were dried over activated alumina from a solvent purification system (SPS) based upon the Grubbs design and de-gassed before use. Glassware was dried for 12 hours prior to use at 120 °C. Benzene-d_6 was de-gassed and stored over 3 Å molecular sieves before use. All reagents were acquired from Sigma Aldrich, Fluorochem or Alfa Aesar and used without further purification unless specified. Where liquids at 25 °C, reagents were dried over activated 3 Å molecular sieves and freeze-pump-thaw degassed prior to use. **1** was prepared following the literature procedure.[3] A range of PTFE products were purchased (Sigma Aldrich, Alfa Aesar) and tested, as per the manuscript, but the optimised reaction is with 1μm particle size PTFE purchased from Sigma Aldrich, used as purchased without any pre-treatment. PVDF was purchased from Sigma Aldrich.

2. Synthetic Procedures

2.1. Defluorination of PTFE with 1 and 4-(dimethylamino)pyridine (DMAP)

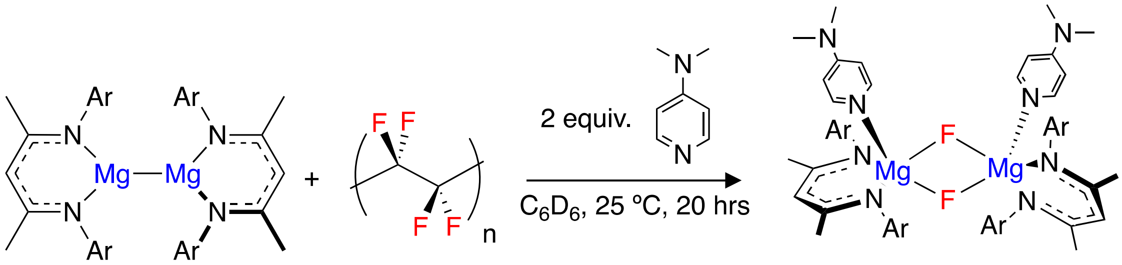

1
Ar = 2,4,6-Me-C_6H_2

8 equiv.
(repeat unit)

2, 85 %

Scheme S1: Defluorination of PTFE with **1** + DMAP.

NMR Scale:

In an N_2 filled glovebox, 10 mg (0.014 mmol) of **1** and 0.028 mmol of DMAP (140 µL of 0.2 M C_6D_6 stock solution) was dissolved in 0.5 mL of C_6D_6, added to a J. Young NMR tube equipped with a ferrocene capillary internal standard, and a t=0 ^{1}H NMR spectrum was recorded. The NMR tube was taken back into the glovebox and PTFE (10 mg, 0.1 mmol repeat unit) was added. The J. Young tube was inverted multiple times and left overnight. After 20 hours, the solution had gone from red to dark brown/red, while the PTFE powder had gone from white to a dark grey. ^{1}H and ^{19}F NMR spectra were recorded. The 85 % yield of the product **2** was determined *in situ* by integral comparison to the ferrocene internal standard in the ^{1}H NMR spectrum. This yield was cross-referenced by ^{19}F NMR spectroscopy, through addition of a fluorinated internal standard (1,2-difluorobenzene) at the end of the reaction, and comparison of the integral to that of **2**.

Preparative Scale:

In an N_2 filled glovebox, 500 mg (0.7 mmol) of **1** and 171 mg (1.4 mmol) of DMAP were added to a Schlenk flask (with no stirrer bar) and dissolved in 10 mL of C_6H_6. PTFE (500 mg, was then added. The flask was manually swirled for 15 seconds and left overnight. After 16 hours, the solution had gone from red to dark brown/red. The C_6H_6 solution was separated from the solid by cannula filtration. The product **2** could be isolated by removal of the solvent *in vacuo*, in an 85 % yield (591 mg, 0.6 mmol). Single-crystals suitable for X-ray crystallography were grown by slow evaporation of a concentrated C_6H_6 solution of **2**. The dark grey solid polymer product is isolated after cannula filtration, and washed with toluene (3x 5 mL) and hexane (3 x 15 mL), and dried under vacuum.

Characterisation of 2:

^{19}F NMR (C_6D_6, 100 MHz, 298 K): δ -183.9 (s, Mg–**F**).

^{1}H NMR (C_6D_6, 400 MHz, 298K): 1.77 (s, 12H, NCC**H$_3$**), 2.01 (s, 24H, ortho-C**H$_3$**), 2.20 (s, 12H, DMAP N(C**H$_3$**)$_2$), 2.33 (s, 12H, para-C**H$_3$**), 5.02 (s, 2H, CH_3C(C**H**)CCH_3), 6.04 (d, 4H, $^3J_{H\text{-}H}$ = 4.9 Hz, DMAP Ar-**H**), 6.95 (s, 8H, Ar-C**H**), 8.16 (d, 4H, DMAP Ar-**H**).

^{13}C NMR (C_6D_6, 125 MHz, 298 K): δ 19.15 (ortho-C**C**H_3), 21.31 (para-C**C**H_3), 23.76 (NC**C**H_3), 38.19 (DMAP N(**C**H_3)$_2$), 93.36 (CH_3C(**C**H)CCH_3), 106.28 (DMAP Ar-**C**), 129.01 (Ar-**C**H), 131.20 (C^{IV}), 132.65 (C^{IV}), 148.77 (C^{IV}), 150.28 (DMAP Ar-**C**), 166.17 (N**C**CH_3).

Single Crystal X-Ray Crystallography:

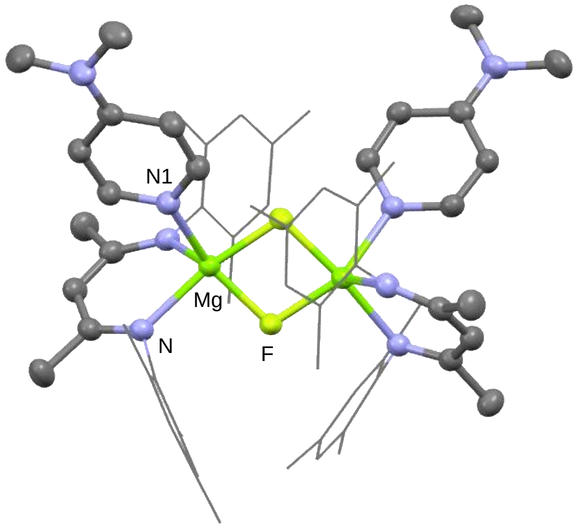

Figure S1: The X-ray crystal structure of **2** (50 % probability ellipsoids). Hydrogen atoms omitted for clarity.

*Crystal data for **2**:* $C_{60}H_{75}F_2Mg_2N_8$, 1.5(C_6H_6), M = 997.92, orthorhombic, Pbcn (no. 60), a = 21.1636(10), b = 22.1040(10), c = 27.3220(2) Å, α = 90, β = 90, γ = 90°, V = 12781.24(13) Å^3, Z = 8, D_c = 1.040 g cm^{-3}, μ(Cu-Kα) = 0.644 mm^{-1}, T = 173 K, clear brownish red plank, Agilent Xcalibur PX Ultra A diffractometer; 12808 independent measured reflections (R_{int} = 0.0332), F^2 refinement, R_1(obs) = 0.0422, wR_2(all) = 0.1310, 10196 independent observed absorption-corrected reflections [$|F_o| > 4\sigma(|F_o|)$, completeness to θ_{full}(147.5°) = 99.9%], 669 parameters. CCDC 2243949. There are 1.5 molecules of disordered C_6H_6 per asymmetric unit which could not be satisfactorily modelled, so consequently were removed using the BYPASS function of Olex2. This accounts for 504 electrons in a volume of 2420 Å^3 per unit cell.

Successive Defluorination Cycles

Successive defluorination cycles were carried out on the same sample of PTFE, as per the procedure described above. After each reaction, **2** was decanted away from the PTFE sample, which was then washed with toluene (3 x 1 mL) and dried under vacuum, before before being re-subjected to **1** + DMAP. We were able to observe the formation of **2** for 7 successive cycles of defluorination. The yield of **2** decreases throughout each successive defluorination, to a minimum of 5 % in the final cycle.

Defluorination Cycle	1	2	3	4	5	6	7
Yield of **2**	85 %	80 %	70 %	17 %	14 %	12 %	5 %

Table S1: Successive defluorination steps and corresponding yields of **2**. Yields determined by addition of fluorobenzene standard at the end of each reaction.

Equivalents of DMAP:

To investigate how the equiv. of DMAP affected the reaction, 3 parallel reactions with both 1, 2 and 10 equiv. of DMAP added per Mg–Mg bond were conducted. While adding 10 equiv. of DMAP gave a very similar outcome to adding 2 equiv., that of the clean formation of **2** in 70 – 80 % yields, adding just 1 equiv. of DMAP led to a slightly different outcome. Two signals are present in the ^{19}F NMR spectrum, [^{Mes}*BDI*Mg(F)]$_3$ at δ -203.5 ppm (^{Mes}*BDI* = (2,4,6-Me-C_6H_2NCMe)$_2$CH),[4] and a signal at δ -192.3 proposed to be the single DMAP coordinated product ^{Mes}*BDI*Mg(F)–Mg(F)(DMAP)*BDI*^{Mes}. Addition of a further equivalent of DMAP resolves this mixture to form only **2** (Scheme S2). As a result, the optimised reaction was considered that when 2 equiv. of DMAP added per Mg–Mg, as this drives the reaction to the thermodynamic product **2**.

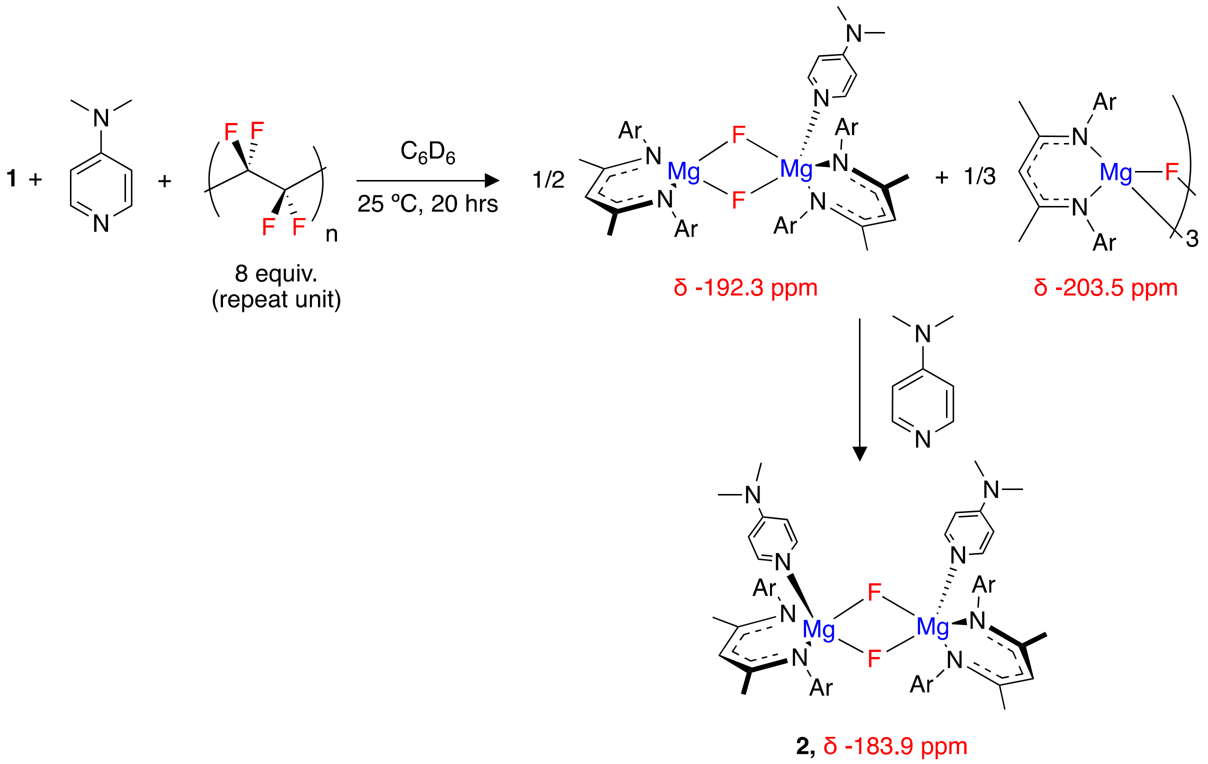

Scheme S2: Demonstration of the requirement for a second equivalent of DMAP.

Stoichiometry of PTFE

The number average molecular weight of PTFE is in the range $10^6 – 10^7$ g mol^{-1}. If we consider the fact that each Mg–Mg unit (with M_W of **1** = 750 g mol^{-1}) can only react with 1 repeat unit of C_2F_4 (M_W = 100 g mol^{-1}), then we can treat PTFE as though it consists of all chains of length C_2F_4. So, in a reaction of 100 mg of **1** with 100 mg of PTFE, we have an effective molar ratio of 0.13 mmol of **1** to 1 mmol PTFE repeat units. This corresponds to 8 equivalents of PTFE repeat unit relative to **1**. Within that, we can consider there are 2 Mg atoms per mmol of **1**, and 4 F atoms per mmol of PTFE repeat unit. Therefore, in this system there is 0.26 mmol of Mg atoms and 4 mmol F atoms. Therefore, with this consideration, there is an excess of 15 F atoms per Mg atom in the reaction mixture. A similar kind of estimation has been used recently in work on poly(vinyl chloride).[5]

2.2. Defluorination of PTFE with 1

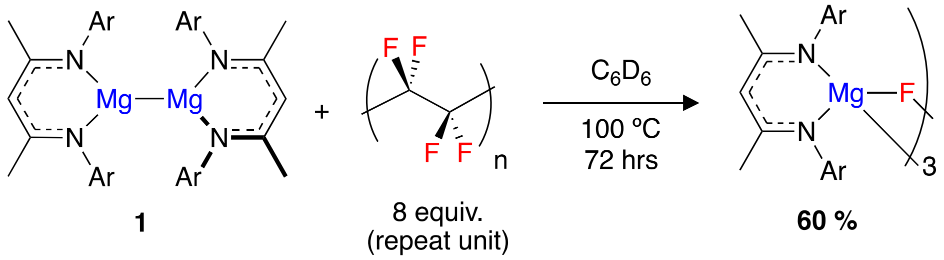

Scheme S3: Defluorination of PTFE with **1**, with no DMAP.

In an N_2 filled glovebox, 10 mg (0.014 mmol) of **1** was dissolved in 0.5 mL of C_6D_6, added to a J. Young NMR tube equipped with a ferrocene capillary internal standard, and a t=0 ^{1}H NMR spectrum was recorded. The NMR tube was taken back into the glovebox and PTFE (10 mg, 0.1 mmol repeat unit) was added. The J. Young tube was inverted multiple times and placed in a 100 °C oil bath for 72 hours. After this time, the solution had gone from pale yellow to dark brown, while the PTFE powder had gone from white to a grey. ^{1}H and ^{19}F NMR spectra were recorded, and the product confirmed to be the previously characterised **2**.[4] A 60 % yield of the product **2** was determined *in situ* by integral comparison to the ferrocene internal standard in the ^{1}H NMR spectrum.

Particle Size Variation Study:

PTFE from different commercial suppliers (Sigma Aldrich, Alfa Aesar), reacted with **1** at 100 °C to form **2**. The reactions were carried out as per the procedure detailed above. Modification of the particle size impacted the recovery of **2** from the mixture. Larger particle sizes of PTFE generally led to lower yields of **2** and longer reaction times. This is reasonable given this reaction is heterogenous and likely to occur at the surface of the polymer.

Particle Size	Supplier	Time	Yield of 2
**1 µm**	Sigma-Aldrich	72 hrs	**60 %**
**6-9 µm**	Alfa Aesar	1 week	**60 %**
**15-25 µm**	Alfa Aesar	1 week	**37 %**
**35 µm**	Sigma-Aldrich	1 week	**24 %**
**200 µm**	Sigma-Aldrich	2 weeks	**33 %**

Table S2: Various PTFE particle sizes and suppliers tested in reaction with **1**.

2.3. Defluorination of poly(vinylidenedifluoride) PVDF with 1 and DMAP

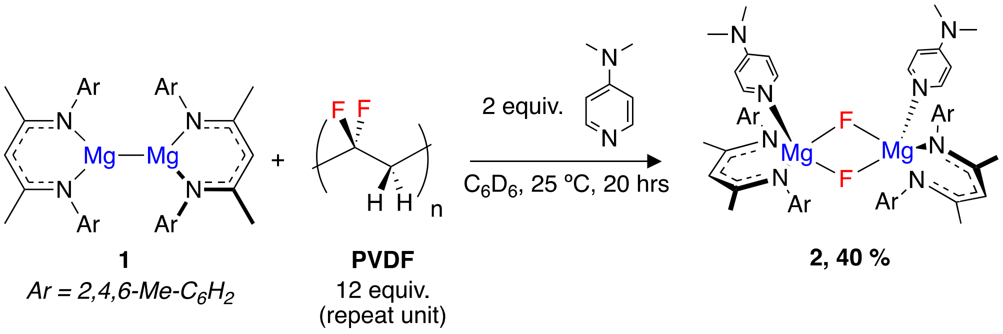

Scheme S4: Defluorination of PVDF with **1** + DMAP.

In an N_2 filled glovebox, 10 mg (0.014 mmol) of **1** and 0.028 mmol of DMAP (140 μL of 0.2 M C_6D_6 stock solution) was dissolved in 0.5 mL of C_6D_6, added to a J. Young NMR tube equipped with a ferrocene capillary internal standard, and a t=0 ^{1}H NMR spectrum was recorded. The NMR tube was taken back into the glovebox and PVDF (10 mg, 0.16 mmol repeat unit) was added. The J. Young tube was inverted multiple times and left overnight. After 20 hours, the solution had gone from red to dark brown/red. ^{1}H and ^{19}F NMR spectra were recorded. The 40 % yield of the product **2** was determined *in situ* by integral comparison to the ferrocene internal standard in the ^{1}H NMR spectrum. This yield was cross-referenced by ^{19}F NMR spectroscopy, through addition of a fluorinated internal standard (1,2-difluorobenzene) at the end of the reaction, and comparison of the integral to that of **2**.

2.4. Defluorination of Perfluoromethylcyclohexane with 1 and DMAP

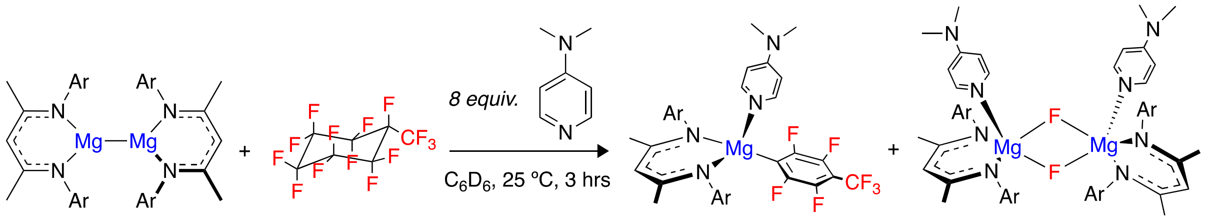

4 equiv.

1

Ar = 2,4,6-Me-C$_6$H$_2$

0.5 equiv.

3, 5-10 %

3.5 equiv.

2, 90 %

Scheme S5: Defluorination of perfluoromethylcyclohexane with **1** and **DMAP**.

In an N_2 filled glovebox, 50 mg (0.07 mmol) of **1** and 17 mg (0.14 mmol) of DMAP were added to a J Young NMR tube and dissolved in 0.5 mL of C_6D_6, and a t=0 ^{1}H NMR spectrum was recorded. The NMR tube was taken back into the glovebox and perfluoromethylcyclohexane was added (3.4 μL, 0.017 mmol). The NMR tube was inverted and left at room temperature for 3 hours. Then, ^{1}H and ^{19}F NMR spectrum were recorded, revealing the formation of **3** and **2**. **3** was characterised by ^{19}F NMR spectroscopy with the fluoroaryl peaks at δ -55.8 (*p*-CF_3), -111.1 (*o*-CF) and -142.2 (*m*-CF). This is similar to a compound previously reported, with THF as the adduct instead of DMAP, with ^{19}F peaks at δ -55.8 (*p*-CF_3), -112.5 (*o*-CF) and -142.2 – -141.8 (*m*-CF).[6] The identity of **3** was verified with its independent synthesis, as per the scheme below.

Independent Synthesis of 3

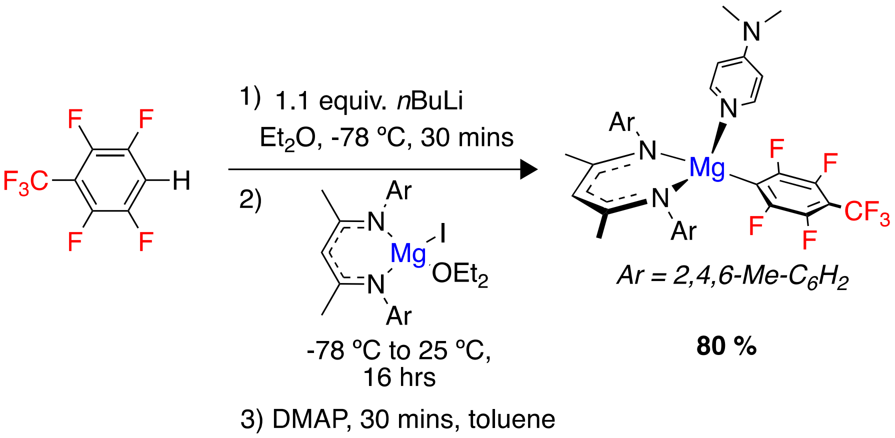

Scheme S6: Independent synthesis of **3**.

To at Et_2O (20 mL) solution of 1,2,4,5-tetrafluorobenzotrifluoride (0.5 mL, 3.67 mmol) cooled to -78 °C, *n*BuLi was added (2.5 mL, 4 mmol). The reaction was stirred for 30 minutes. It is noted here that this reaction must not be heated to room temperature, as the lithiated intermediate is explosive *via* elimination of LiF and formation of benzyne moieties. Instead, at -78 °C an Et_2O solution of ^{Mes}*BDI*Mg(I)(OEt_2) (2.05g, 3.67 mmol) was added. The reaction was stirred at -78 °C for 2 hours, before slowly being warmed to room temperature overnight. The etherate product was isolated as a brown solid by removal of Et_2O *in vacuo* (2.14 g, 90 %). A portion of this sample (100 mg, 0.15 mmol) was re-dissolved in toluene and an equivalent of DMAP was added (19 mg, 0.15 mmol), before removal of solvent *in vacuo* to isolate **3** as a brown solid (99 mg, 0.14 mmol, 95 %).

^{19}**F NMR** (C_6D_6, 100 MHz, 298 K): δ -55.8 (*p*-CF_3), -111.1 (*o*-CF) and -142.2 (*m*-CF).

^{1}**H NMR** (C_6D_6, 400 MHz, 298 K): δ 1.77 (s, 6H, NC(C**H₃**)₂), 2.04 (s, 12H, *o*-MesC**H₃**), 2.16 (s, 6H, DMAP-N(**CH₃**)₂), 2.17 (s, 6H,*p*-MesC**H₃***)*, 5.14 (s, 1H, CH_3C(**CH**)C(CH_3)), 5.82 (DMAP-Ar-**H**), 6.81 (s, 4H, Ar-**H**), 7.94 (DMAP-Ar-**H**).

^{13}**C NMR** (C_6D_6, 125 MHz, 298 K): δ 18.75 (DMAP-N(**C**H_3)$_2$), 20.92 (*p*-Mes**C**H_3), 23.60 (NC(**C**H_3), 38.09 (*o*-Mes**C**H_3), 94.92 (CH_3C(**C**H)CCH_3), 106.63 (DMAP Ar-**C**), 129.52 (Mes-**C**H), 132.01 (Mes-C^{IV}), 133.18 (Mes-C^{IV}), 142.91 (dd, $^{1}J_{CF}$ = 265.0 Hz, Ar-**C**F) 146.15 (Mes-C^{IV}), 149.02 (DMAP Ar^F-**C**), 151.08 (dd, $^{1}J_{CF}$ = 222.2 Hz, Ar^F-**C**F) 168.35 (N**C**CH_3). Note: *ipso*-Ar^F-**C**^{IV}, *para*-Ar^F-**C**^{IV} and **C**F_3 not observed.

Isolated reaction of octafluorotoluene and 1 and DMAP:

In an N_2 filled glovebox, 7 mg (0.01 mmol) of **1** was added to a J Young NMR tube and dissolved in 0.5 mL of C_6D_6, and a t=0 ^{1}H NMR spectrum was recorded. The NMR tube was

taken back into the glovebox and octafluorotoluene was added (2.08 µL, 0.014 mmol). The NMR tube was inverted and left at room temperature for 1 hour. Then, 3 mg (0.02 mmol) of DMAP was added and the reaction left for a further 30 minutes. Then, ^{1}H and ^{19}F NMR spectrum were recorded, revealing the formation of **3** in a 30 % yield, as determined by ^{19}F NMR integral comparison to a fluorobenzene internal standard added at the completion of the reaction. **2** was formed in a 90 % yield, determined by the same method.

2.5. Defluorination of other small-molecule PFAS with 1 and DMAP

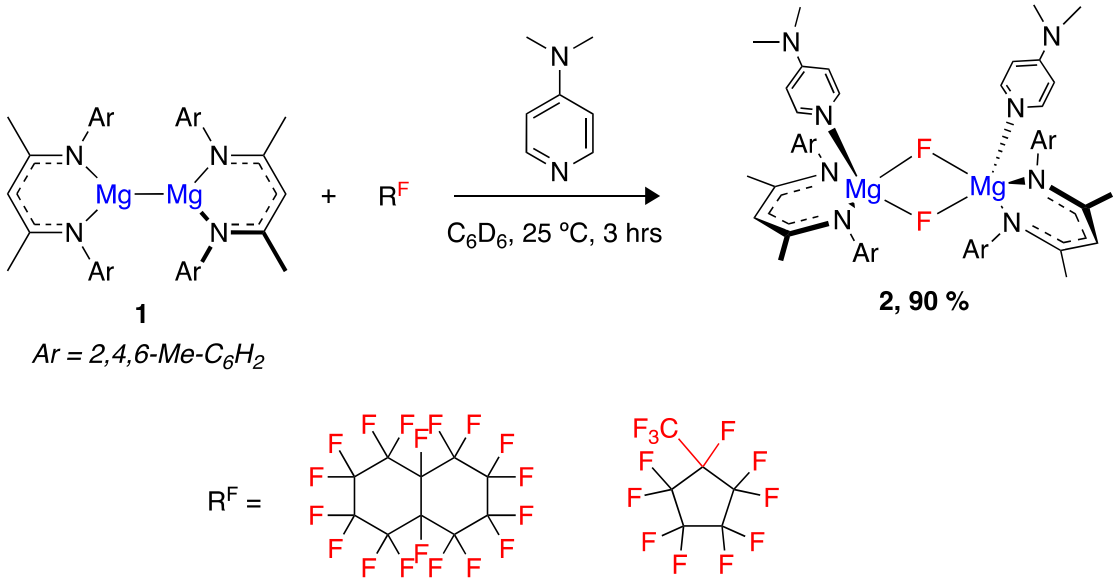

Scheme S7: Defluorination of small-molecule PFAS to form **2-(DMAP$_2$).**

Perfluorodecalin:

In an N_2 filled glovebox, 50 mg (0.07 mmol) of **1** and 17 mg (0.14 mmol) of DMAP were added to a J Young NMR tube and dissolved in 0.5 mL of C_6D_6, and a t=0 ^{1}H NMR spectrum was recorded. The NMR tube was taken back into the glovebox and perfluorodecalin was added (3.2 µL, 0.013 mmol). The NMR tube was inverted and left at room temperature for 3 hours. Then, ^{1}H and ^{19}F NMR spectrum were recorded, revealing the formation of **2** in 90% yield, determined *in situ* by integral comparison to the ferrocene internal standard in the ^{1}H NMR spectrum.

Perfluoromethylcyclopentane:

In an N_2 filled glovebox, 50 mg (0.07 mmol) of **1** and 17 mg (0.14 mmol) of DMAP were added to a J Young NMR tube and dissolved in 0.5 mL of C_6D_6, and a t=0 ^{1}H NMR spectrum was recorded. The NMR tube was taken back into the glovebox and perfluoromethycyclopentane

was added (5.8 µL, 0.033 mmol). The NMR tube was inverted and left at room temperature for 3 hours. Then, ^{1}H and ^{19}F NMR spectrum were recorded, revealing the formation of **2** in 90% yield, determined *in situ* by integral comparison to the ferrocene internal standard in the ^{1}H NMR spectrum.

2.6. Isolation of 1-(DMAP)$_2$

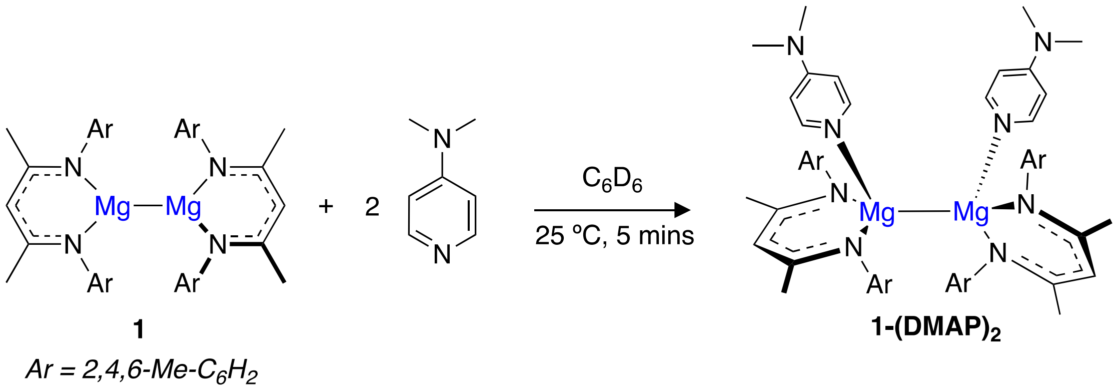

Scheme S8: Isolation of **1-(DMAP)$_2$.**

In a N_2 filled glovebox, **1** (50 mg, 0.07 mmol) was dissolved in toluene (3 mL) in a scintillation vial, and 2 equiv. of DMAP added (0.14 mmol, 17 mg). The reaction was stirred for 5 minutes before removal of the solvent *in vacuo*. Single-crystals were grown suitable for X-ray analysis by slow diffusion of *n*-hexane into a concentrated toluene solution of **1-(DMAP)$_2$**.

1-(DMAP)$_2$ has been previously characterised by NMR and X-ray crystallography, and our data is in agreement.[7]

2.7. Isolation of 1-(DMAP)

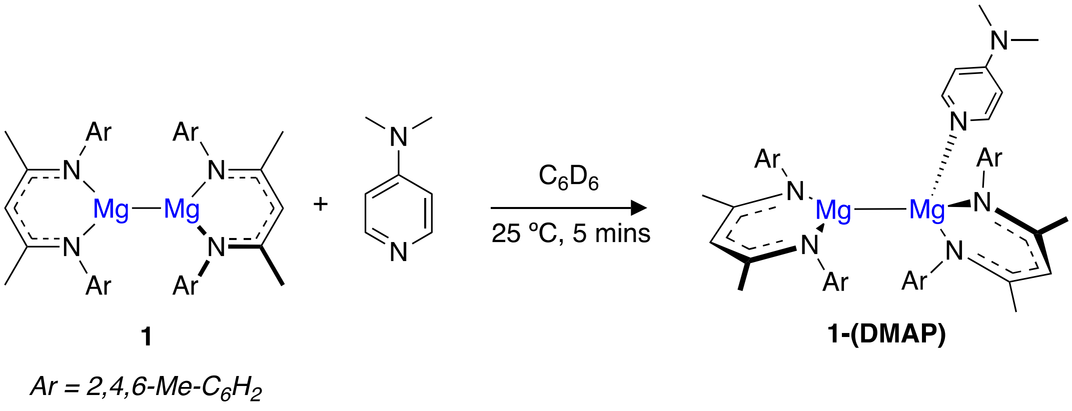

Scheme S9: Isolation of **1-(DMAP).**

In a N_2 filled glovebox, **1** (50 mg, 0.07 mmol) was dissolved in toluene (3 mL) in a scintillation vial, and 1 equiv. of DMAP added (0.07 mmol, 8.6 mg). The reaction was stirred for 5 minutes before removal of the solvent *in vacuo*. Single-crystals were grown suitable for X-ray analysis by slow diffusion of *n*-hexane into a concentrated toluene solution of **1-(DMAP)**.

1-(DMAP):

^{1}H NMR (C_6D_6, 400 MHz, 298K): 1.68 (s, 12H, NCC**H**$_3$), 1.99 (s, 24H, ortho-C**H**$_3$), 2.17 (s, 12H, DMAP N(C**H**$_3$)$_2$), 2.33 (s, 12H, para-C**H**$_3$), 4.93 (s, 2H, CH_3C(C**H**)CCH_3), 6.00 (d, 4H, DMAP Ar-**H**), 6.92 (s, 8H, Ar-C**H**), 8.02 (d, 4H, DMAP Ar-**H**).

Small brown-red plank crystals were successfully mounted but were found to be weakly diffracting, resulting in generally weak data, especially for the high angle diffraction peaks. Nevertheless, connectivity data could be established with an acceptable R_1(obs) value of 8.87% (but a high R_{int} value of 17.21%), confirming the structure of **1-(DMAP)** that had co-crystallised with **1-(DMAP)$_2$**. The crystal was found to contain two independent molecules of compound **1** which differ by the number of coordinated DMAP units per dimer (1 and 2, respectively).

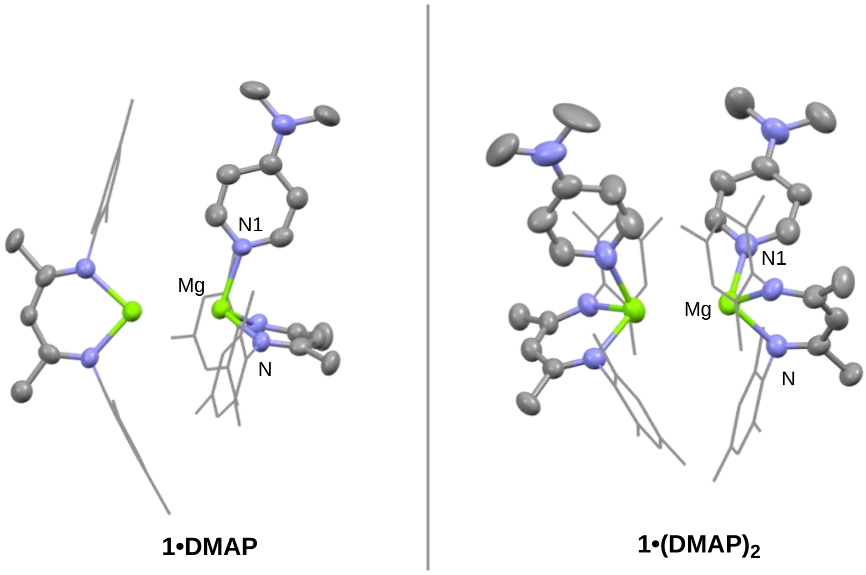

Figure S2: The X-ray crystal structures of the co-crystallised **1-(DMAP)** and **1-(DMAP)$_2$**, with the unit cell for each molecule displayed separately for clarity (50 % probability ellipsoids). Hydrogen atoms omitted for clarity.

*Crystal data for **1-(DMAP)_1-(DMAP)$_2$**:* $C_{53}H_{68}Mg_2N_6$, $C_{60}H_{79}Mg_2N_8$, 3.2(C_7H_8), M = 1982.94, triclinic, P-1 (no. 2), a = 12.7599(8), b = 22.0074(15), c = 23.8871(17) Å, α = 97.926(6), β = 104.900(6), γ = 97.110(5)°, V = 6330.9(8) Å^3, Z = 2, D_c = 1.037 g cm^{-3}, μ(Cu-Kα) = 0.691 mm^{-1}, T = 173 K, clear colourless block, Agilent Xcalibur PX Ultra A diffractometer; 24125

independent measured reflections (R_{int} = 0.1721), F^2 refinement, R_1(obs) = 0.0887, wR_2(all) = 0.2731, 6028 independent observed absorption-corrected reflections [$|F_o| > 4\sigma(|F_o|)$, completeness to θ_{full}(147.8°) = 98.4%], 1346 parameters.

3. Reactions of 2 with Electrophiles

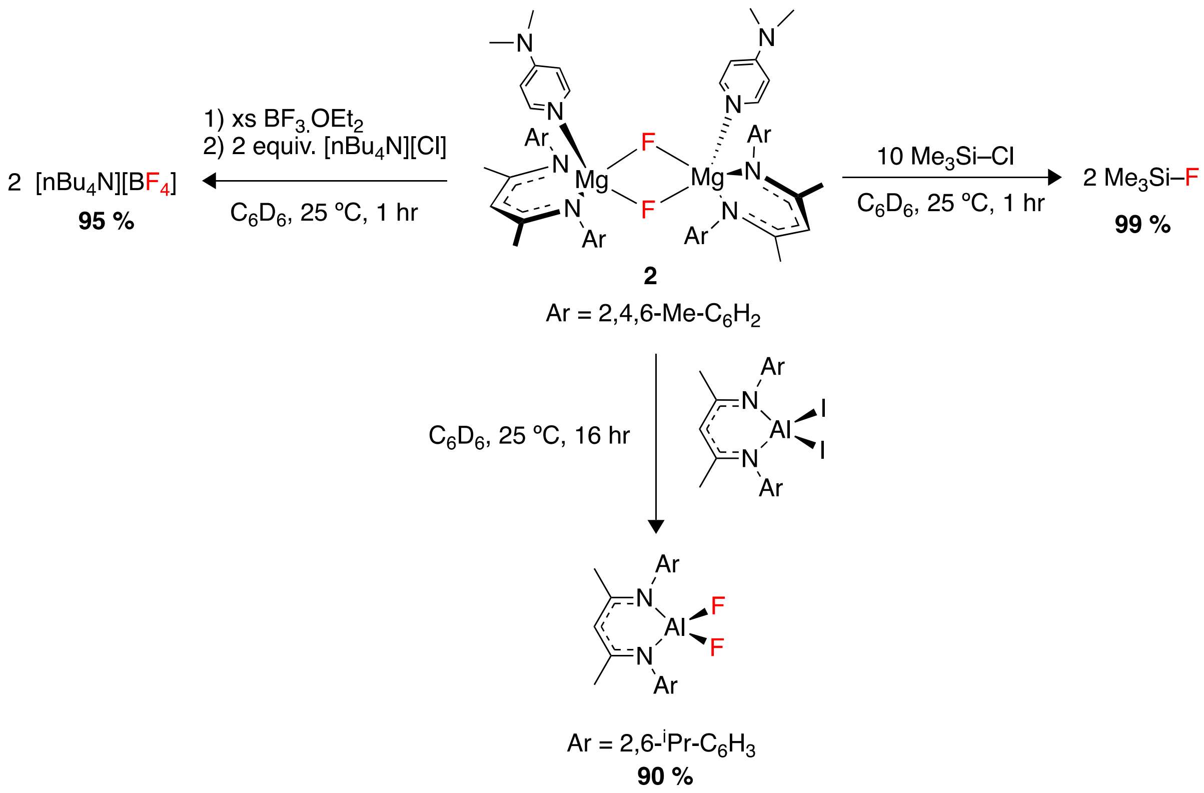

Scheme S10: Reactions of **2** with electrophiles.

Reaction with Trimethylsilyl chloride:

In an N_2 filled glovebox, 10 mg of **2** (0.01 mmol) was dissolved in C_6D_6 and added to a J Young NMR tube, 1,2-difluorobenzene was added as an internal standard (0.002 mmol), along with an external standard capillary tube of ferrocene in C_6D_6. ^{1}H and ^{19}F spectra were recorded. To this NMR tube, Me_3Si–Cl (12.7 μL, 0.1 mmol) was added. After 1 hour, ^{1}H and ^{19}F spectra were recorded, and the product Me_3SiF was formed in a quantitative yield, whose data matched those reported in the literature.[8]

Me_3SiF:

^{19}F NMR (C_6D_6, 100 MHz, 298K): -157.2 (sept, $^3J_{HH}$ = 7.0 Hz).

^{1}H NMR (C_6D_6, 400 MHz): 0.11 (d, $^3J_{HH}$ = 7.0 Hz).

Reaction with BF_3:

In an N_2 filled glovebox, 10 mg of **2** (0.01 mmol) was dissolved in C_6D_6 and added to a J Young NMR tube, along with an standard capillary tube of fluorohexane in C_6D_6. ^{1}H and ^{19}F spectra were recorded. To this NMR tube, $BF_3.OEt_2$ (6.2 μL, 0.05 mmol, 5 equiv.) was added. After 1 hour, ^{1}H and ^{19}F spectra were recorded, to ensure all of **2** had reacted. At this point, [*n*-Bu_4N][Cl] was added (5.6 mg, 0.02 mmol). After 1 hour, ^{1}H and ^{19}F spectra were recorded,

confirming the formation of [n-Bu_4N][BF_4]. All the volatiles were removed *in vacuo* and the sample was re-dissolved in CD_2Cl_2. 1,2-difluorobenzene (2 uL) internal standard was added and ^{1}H, ^{11}B, and ^{19}F spectra recorded, showing formation of [nBu_4N][BF_4] in a 95 % yield. Data for [n-Bu_4N][BF_4] in very close agreement with literature.[9] A control reaction between $BF_3.OEt_2$, DMAP and [n-Bu_4N][Cl] in CD_2Cl_2 did not lead to formation of [n-Bu_4N][BF_4].

[*n*-Bu_4N][BF_4]:

^{19}F NMR (CD_2Cl_2, 100 MHz, 298 K): δ -151.7 (br)

^{11}B NMR (CD_2Cl_2, 128 MHz, 298 K): δ 1.13 (br)

Also present in the reaction mixture is BF_3•DMAP, ^{19}F NMR (CD_2Cl_2, 100 MHz, 298 K): δ -151.8 (q, $^{1}J_{BF}$ = 13.7 Hz); ^{11}B NMR (CD_2Cl_2, 128 MHz, 298 K): δ 0.26 (q, $^{1}J_{BF}$ = 13.7 Hz).

Reaction to form aluminium fluoride:

In an N_2 filled glovebox, 10 mg of **2** (0.01 mmol) was dissolved in C_6D_6 and added to a J Young NMR tube, 1,2-difluorobenzene was added as an internal standard (0.002 mmol), along with an external standard capillary tube of ferrocene in C_6D_6. ^{1}H and ^{19}F spectra were recorded. To this NMR tube, ^{Dipp}*BDI*AlI_2 (^{Dipp}*BDI* = (2,6-(*i*-Pr)-$C_6H_3NCMe)_2CH$) (7 mg, 0.01 mmol) was added. After 1 hour, ^{1}H and ^{19}F spectra were recorded, and the product ^{Dipp}*BDI*AlF_2 was formed in a quantitative yield, with data matching that as reported in the literature.[10] ^{Dipp}*BDI*AlF_2 has been shown in a previous study as a reagent for synthesising acyl fluorides.[10]

^{Dipp}*BDI*AlF_2:

^{1}H NMR (C_6D_6, 400 MHz, 298K): δ 1.10 (d, 12H, CH(C**H**$_3$)$_2$, $^{3}J_{HH}$ = 6.8 Hz), 1.42 (d, 12H, CH(C**H**$_3$)$_2$, $^{3}J_{HH}$ = 6.8 Hz), 1.53 (s, 6H, C**H**$_3$), 3.31 (sept, 4H, C**H**(CH_3)$_2$, $^{3}J_{HH}$ = 6.8 Hz), 4.94 (s, 1H, C(CH_3)C**H**C(CH_3)), 7.05-7.15 (m, 6H, Ar**H**)

^{19}F NMR (376.5 MHz, C_6D_6, 298 K): δ -173.6 (2F, Al**F**)

4. Solid-State Characterisation of PTFE-R

PXRD:

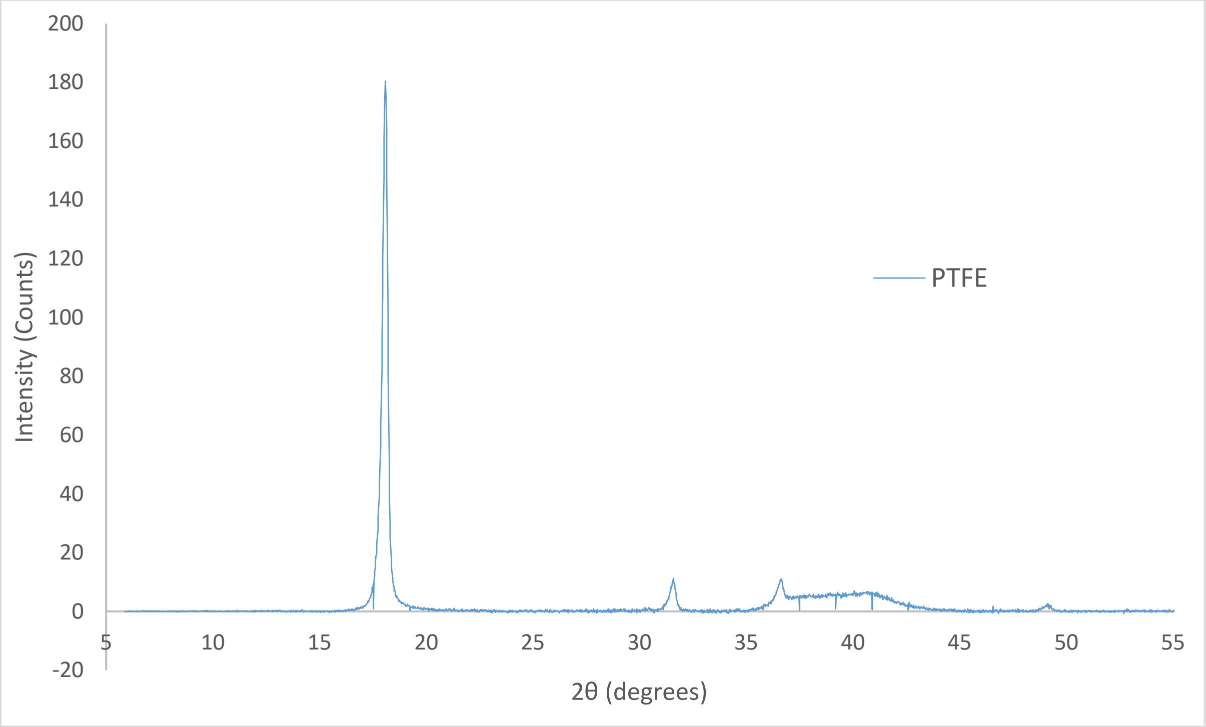

Figure S3: PXRD spectrum for **PTFE**.

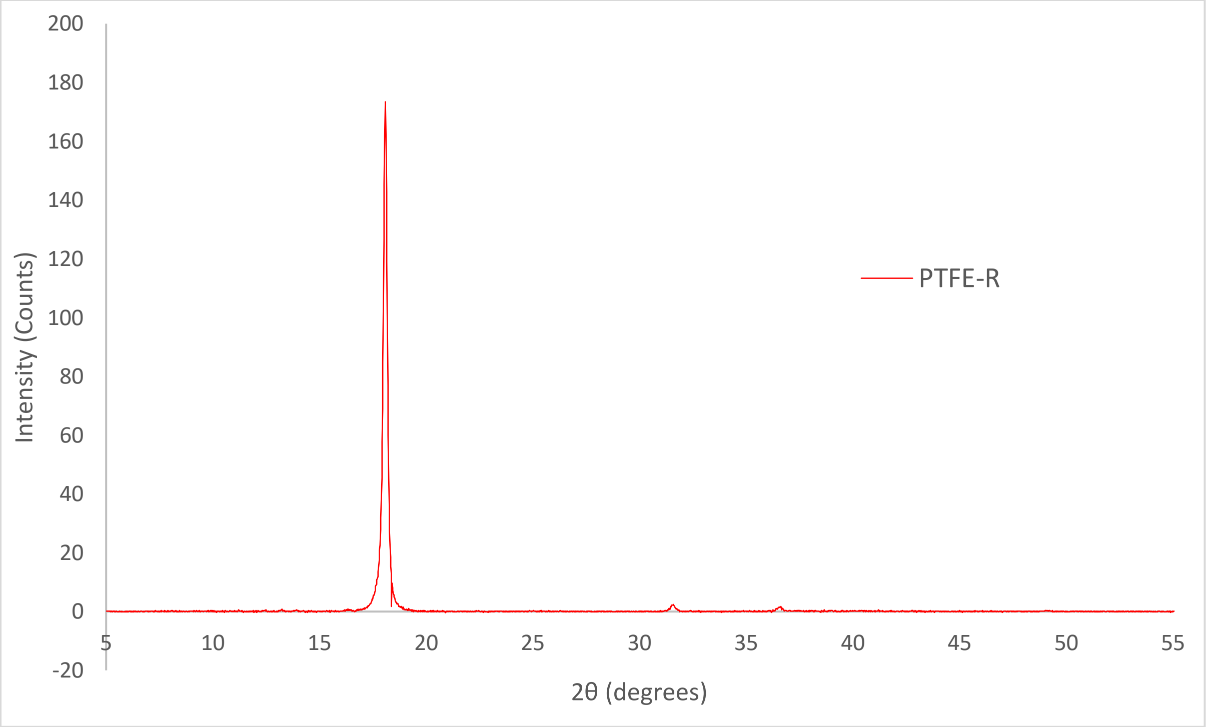

Figure S4: PXRD spectrum for **PTFE-R**.

IR:

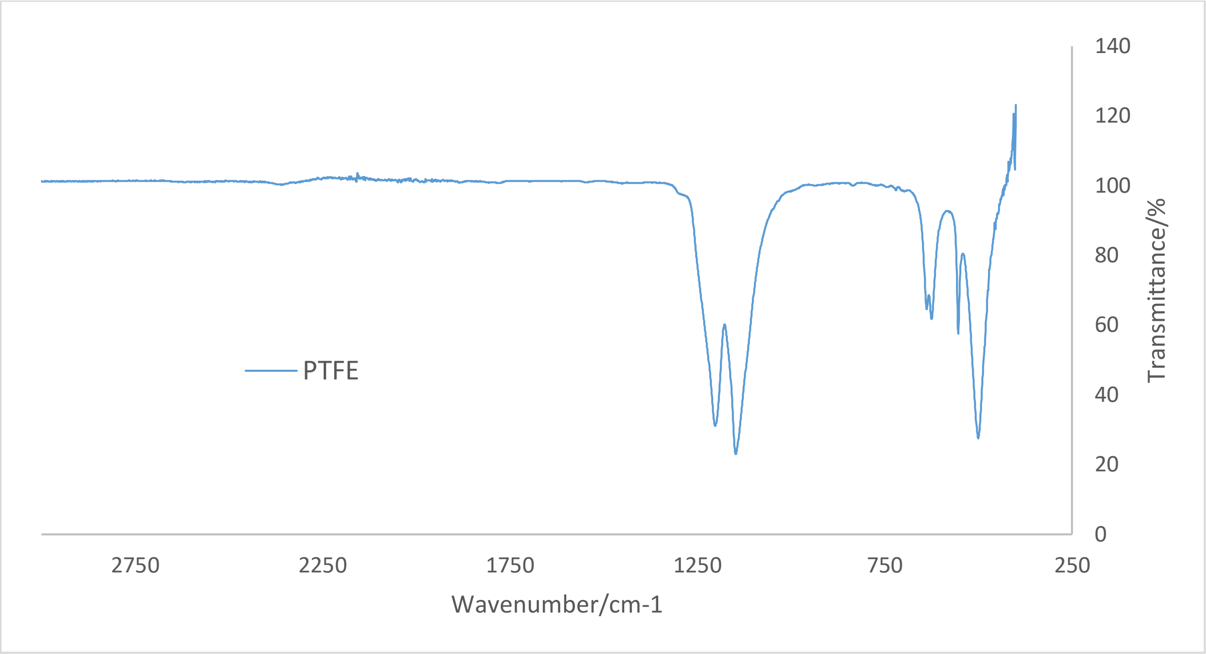

Figure S5: IR spectra for **PTFE**.

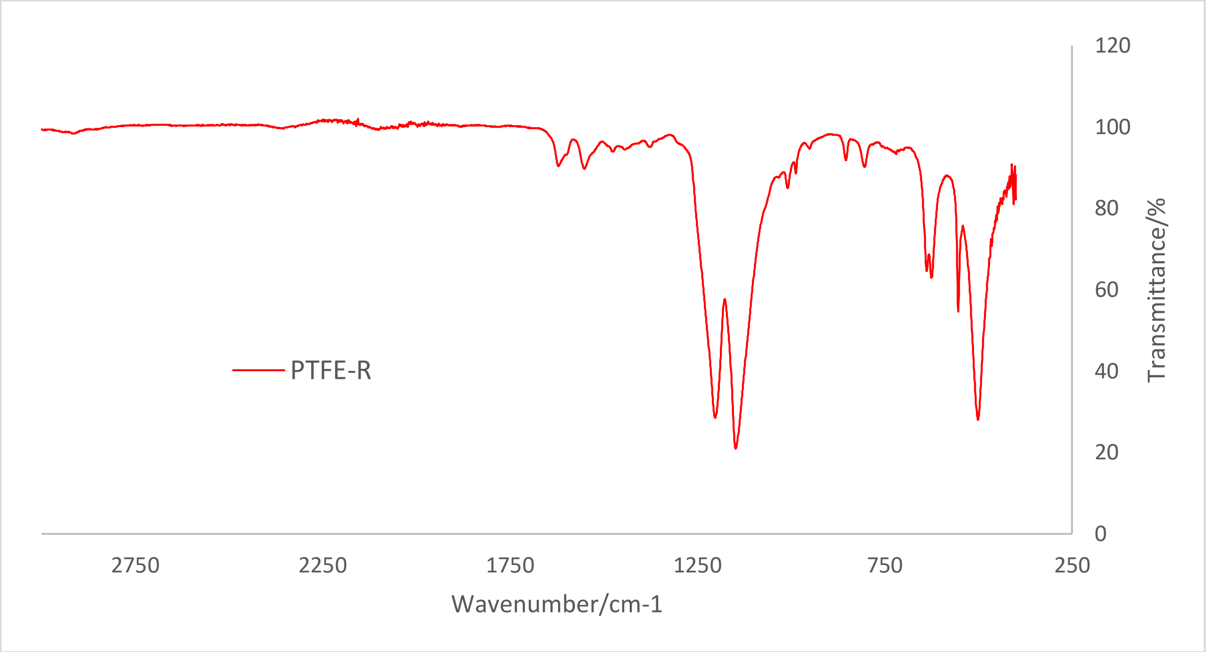

Figure S6: IR spectra for **PTFE-R**.

Reaction of PTFE-R with BH_3.THF:

A sample of **PTFE-R** (50 mg, 0.5 mmol of repeat unit if assume repeat units are C_2F_4) was reacted with an excess of BH_3·THF (1 mmol, 1 mL of 1.0 M in THF) for 72 h (no stirrer bar, to avoid any PTFE contamination). The polymer was isolated by filtration, washing with toluene (3 x 5 mL) and drying under vacuum. The polymer was then analysed by IR spectroscopy, and it was observed that the key vibrations assigned to C=C modes are no longer present (as would be expected from hydroboration of the C=C functional group).

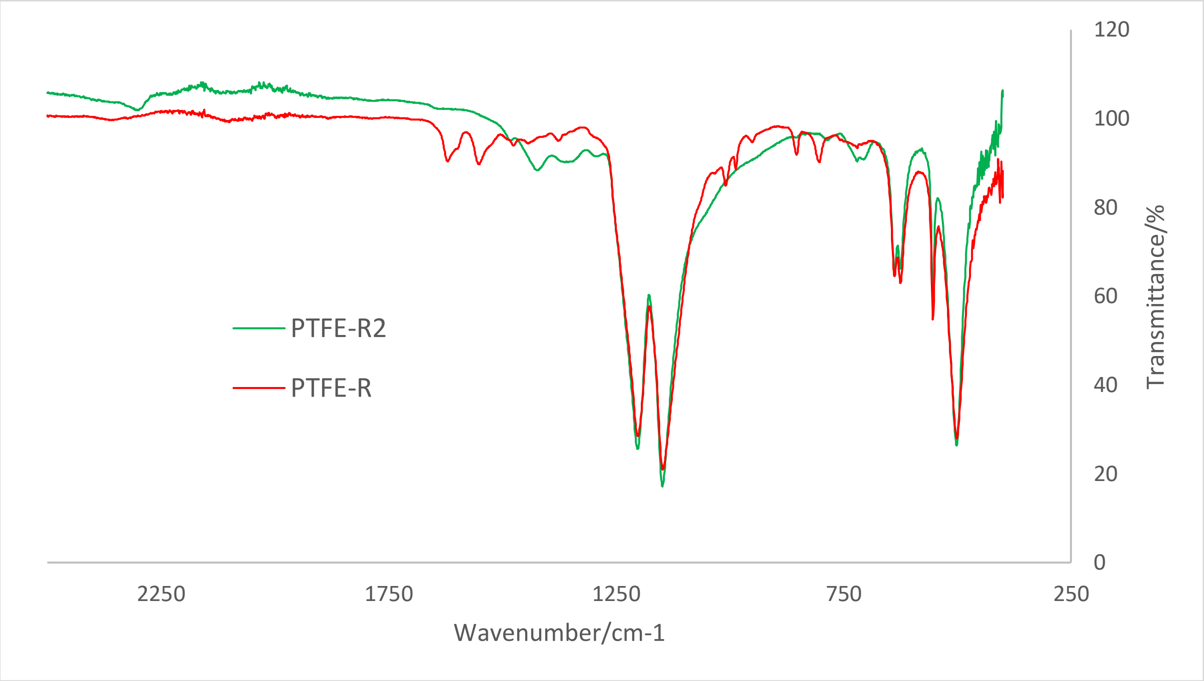

Figure S7: IR spectra for the hydroborated polymer **PTFE-R2**, and comparison to **PTFE-R**.

XPS:

Data is shown below for the C(1s) and F(1s) XPS scan of **PTFE** and **PTFE-R**.

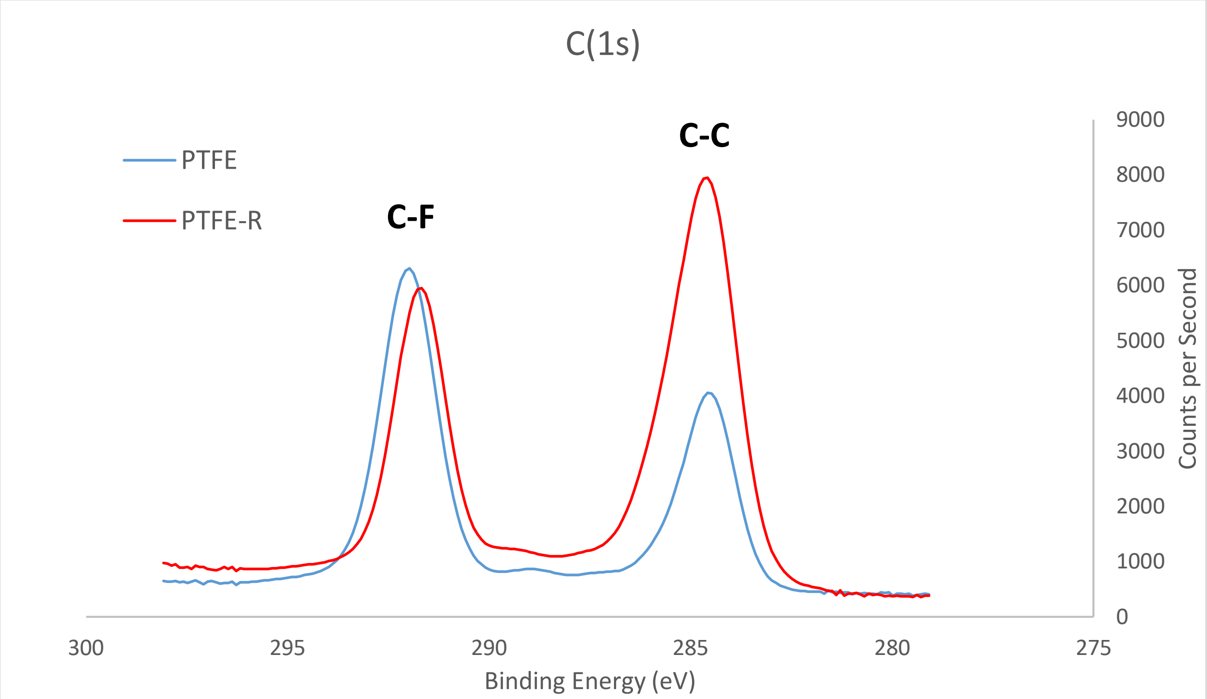

Figure S8: XPS C(1s) scan of **PTFE** and **PTFE-R**.

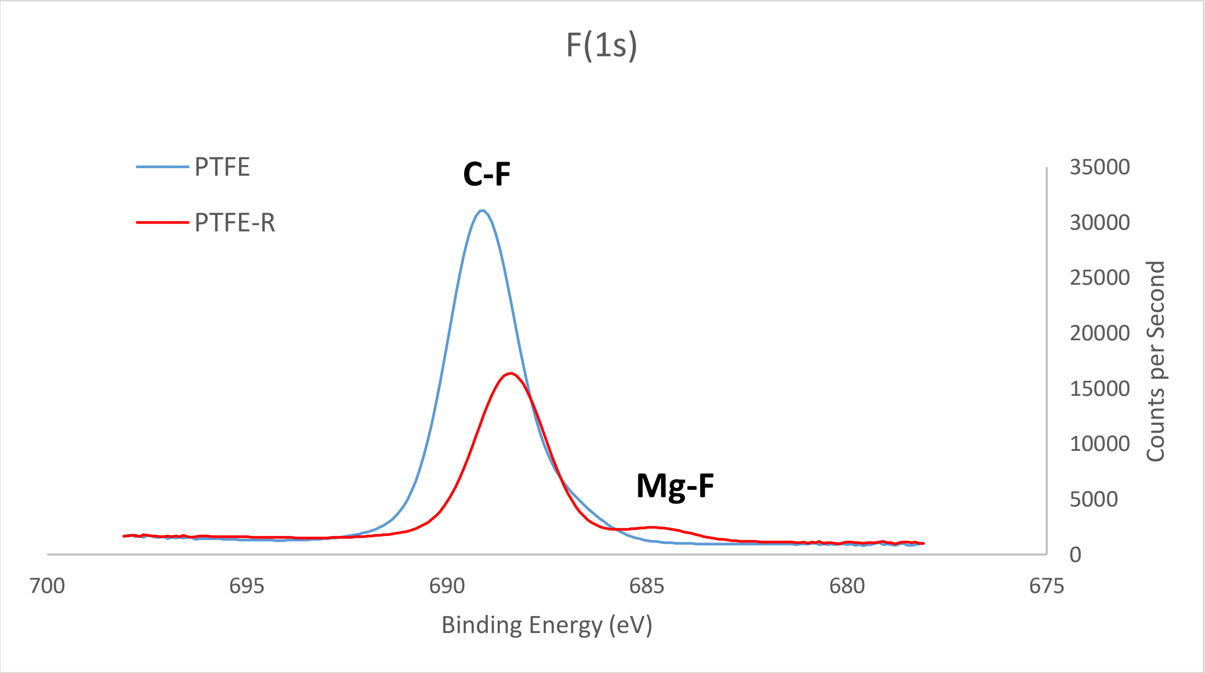

Figure S9: XPS F(1s) scan of **PTFE** and **PTFE-R**.

SEM:

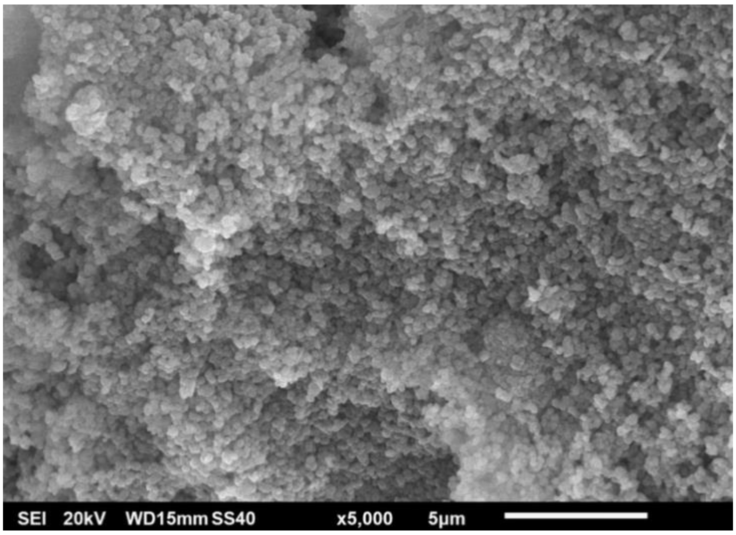

Figure S10: SEM image of **PTFE** at x 5000 magnification

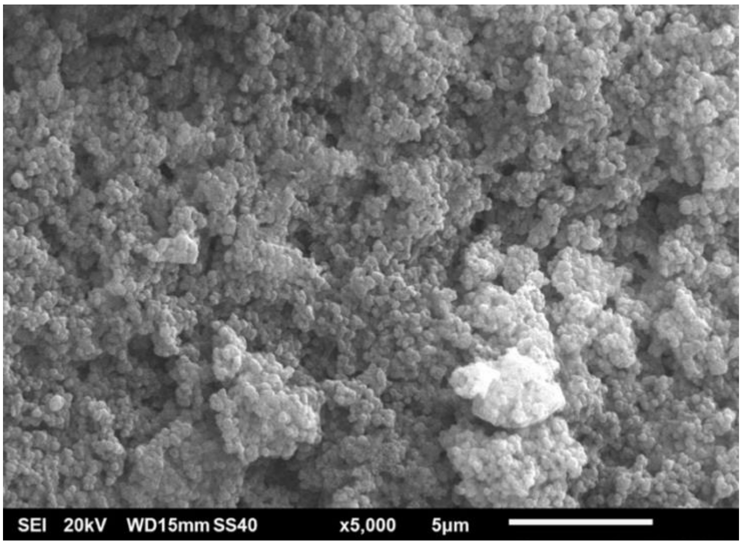

Figure S11: SEM image of **PTFE-R** at x 5000 magnification

ssNMR:

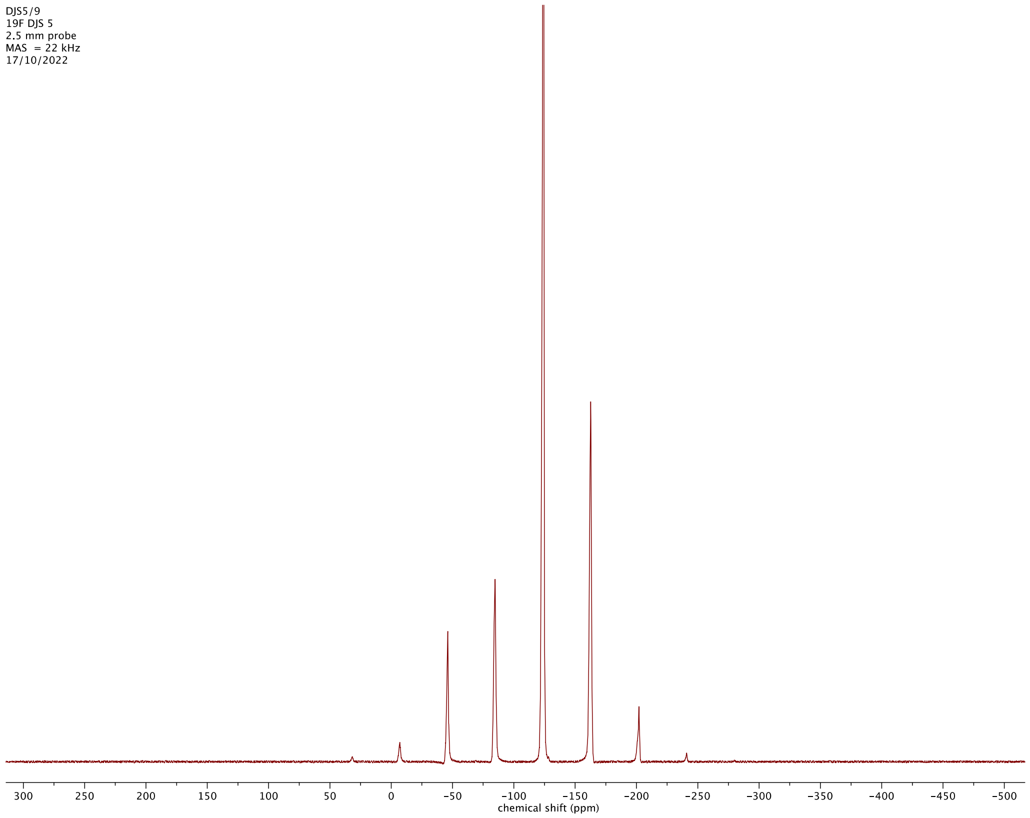

Figure S12: MAS-NMR ^{19}F spectrum of PTFE.

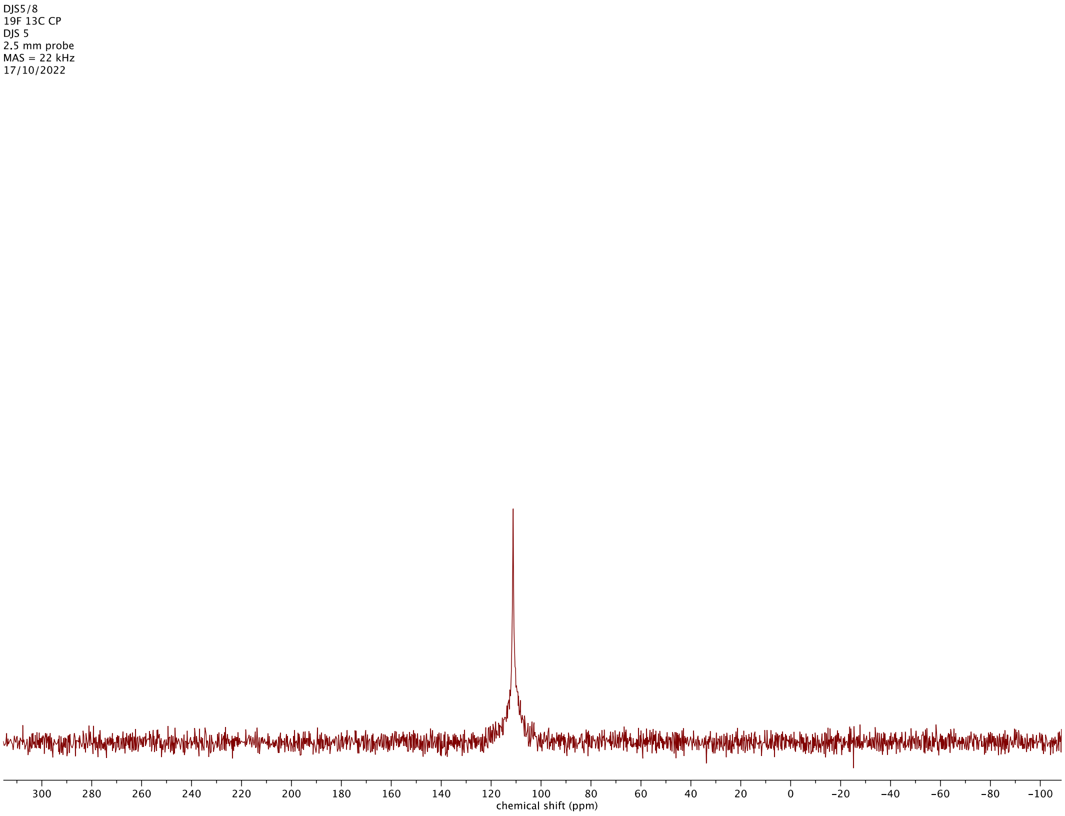

Figure S13: MAS-NMR ^{19}F-^{13}C Cross Polarisation spectrum of PTFE.

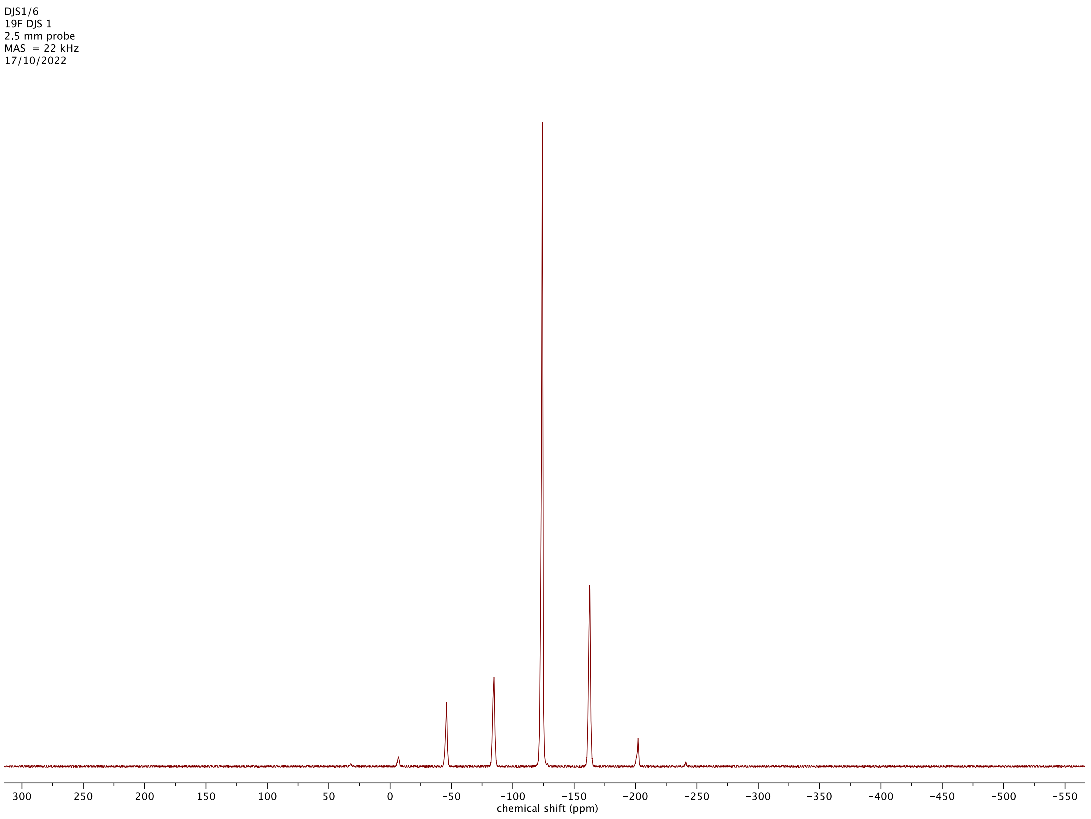

Figure S14: MAS-NMR ^{19}F spectrum of PTFE-R.

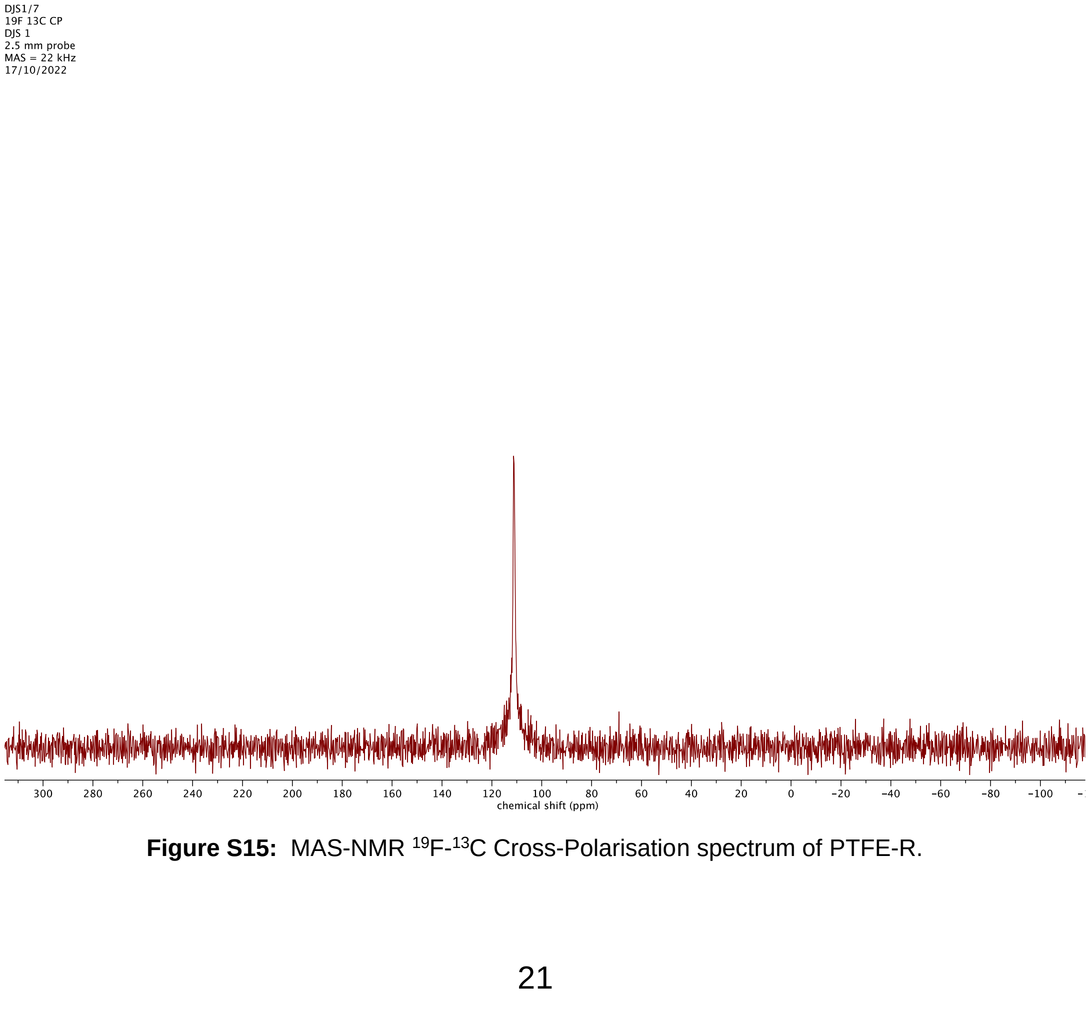

Figure S15: MAS-NMR ^{19}F-^{13}C Cross-Polarisation spectrum of PTFE-R.

5. Computational Methods

DFT calculations were run using Gaussian 09 (Revision D.01)[11] using the using the B3PW91 density functional,[12–16] and an ultrafine integration grid (keyword int=ultrafine).[17] Geometry optimisations and frequency calculations were carried out using BS1, while single point frequency calculations were then carried out at BS2 to obtain the final free energies.[18]

BS1 was built as follows. Mg centres were described with Stuttgart SDDAll RECPs and associated basis sets, while a hybrid basis set was used for the other atoms: 6-31g**(C, H)/6-311+g*(N, F).

BS2 was built as follows. Mg centres were described with Stuttgart SDDAll RECPs and associated basis sets, while 6-311+g* was used for all other atoms.

BS3 was built as follows. Mg centres were described with Stuttgart SDDAll RECPs and associated basis sets, while 6-311++g* was used for all other atoms.

BS4 was built as follows. Mg centres were described with Stuttgart SDDAll RECPs and associated basis sets, while Ahlrichs triple-ξ basis set def2-TZVPP was used for all other atoms.[19]

Geometry optimisation calculations were performed without symmetry constraints. The Gaussian 09 default optimisation criteria were tightened to 10^{-9} on the density matrix and 10^{-7} on the energy matrix. The default numerical integration grid was also improved using a pruned grid with 99 radial shells and 590 angular points per shell. Frequency analyses for all stationary points were performed using the enhanced criteria to confirm the nature of the structures as either minima (no imaginary frequency) or transition states (only one imaginary frequency). Single point solvent corrections (benzene, ε = 2.2706) were applied using the polarizable continuum model (PCM) to free energies.[20] Single-point dispersion corrections using Grimme's D3 correction were applied to free energies, with Becke-Johnson damping applied for the B3PW91 functional.[21,22] Intrinsic reaction coordinate (IRC) calculations followed by full geometry optimisations on final points were used to connect transition states and minima located on the potential energy surface allowing a full energy profile (calculated at 298.15 K, 1 atm) of the reaction to be constructed.[23,24] The graphical user interface used to visualise the various properties of the intermediates and transition states was GaussView 5.0.9.[25] Natural Bond Orbital analysis was carried out using NBO 6.0.[26]

5.1. Discussion of Computational Model

DMAP binding to **1** is assumed to be fast and reversible. This assumption is reinforced by the ^{1}H NMR spectroscopic data at 25 °C of **1-(DMAP)** which reveals a broadened set of signals corresponding to 1 symmetrical ligand environment, demonstrating the fluxional behaviour of DMAP, where it is rapidly moving between Mg centres. Previous work by the Jones group came to the same conclusion.[27]

In the reaction of **1** with PTFE, 2 equiv. of DMAP are used, as this drives the reaction to the thermodynamic product of **2.** However, the reaction of **1** and PTFE also proceeds at room temperature when only 1 equiv. of DMAP is added. This reaction formed a mixture of products, which was resolved to form the singular thermodynamic product of **2** by the addition of a second equiv. of DMAP.

Hence in the computational model, we consider **1-(DMAP)** as the active species and zero-energy point for the reaction with C_2F_6. The assumption is that DMAP can transfer to a different magnesium species with an insignificant energy penalty generating **1-(DMAP)** in situ. Calculations on a series of isodesmic reactions that suggest that DMAP can exchange between Mg centres in **1** and **2** with only a small energy penalty (Scheme S11). This model is more appropriate than considering the formal association and dissociation of DMAP to Mg atoms, as this method assumes DMAP can be free in solution, which is highly unlikely in practise, and occurs a significant and unrealistic energy penalty (~ 10 kcal mol^{-1}).

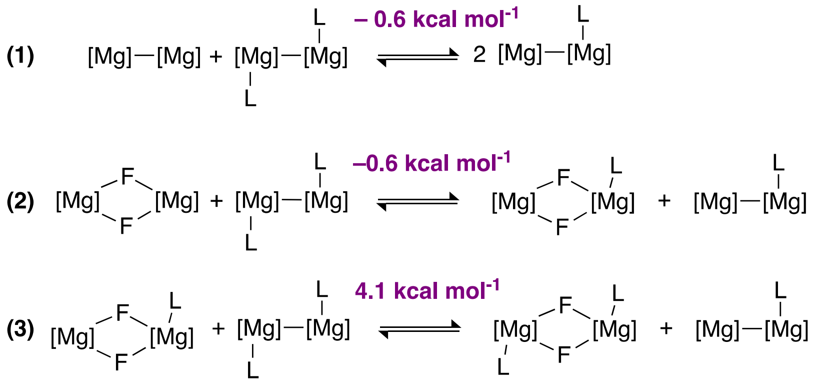

Scheme S11: Equilibria demonstrating the ease of DMAP transfer between different magnesium species present in the reaction mixture.

5.2. Alternative Mechanisms

After establishing the lowest-energy transition state for C–F cleavage of C_2F_6 with **1-(DMAP)**, we calculated similar transition states for the same bond breaking by **1** and **1-(DMAP)$_2$** (Figure S16). Attack of **1** at C_2F_6 was calculated *via* **TS-1A** ($\Delta G^{\ddagger}_{298K}$ = 30.2 kcal mol^{-1}), and by **1-(DMAP)$_2$** *via* **TS-1B** ($\Delta G^{\ddagger}_{298K}$ = 38.2 kcal mol^{-1}), both giving higher activation energy barriers that the reaction of **1-(DMAP)** with C_2F_6.

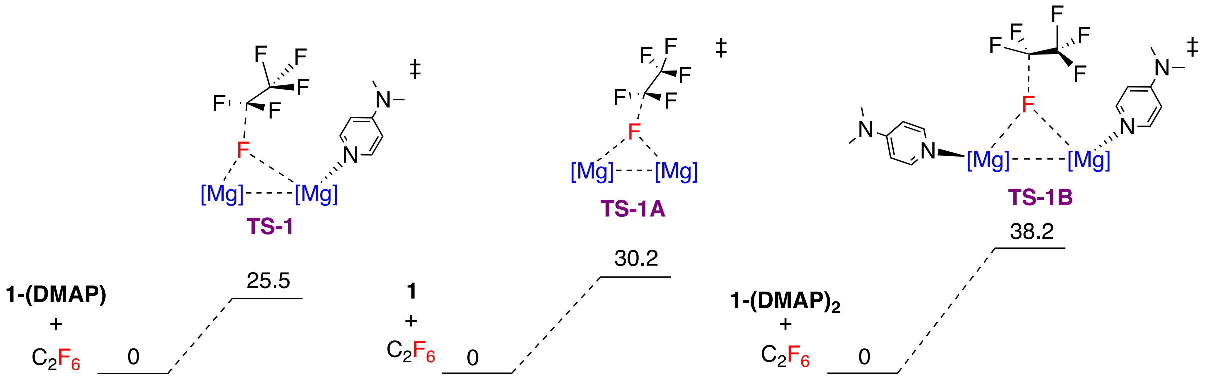

Figure S16: Calculated Potential Energy Surfaces for C–F cleavage of C_2F_6 by **1 (TS-1A)** vs **1-(DMAP) (TS-1)** vs **1-(DMAP)$_2$ (TS-1B).**

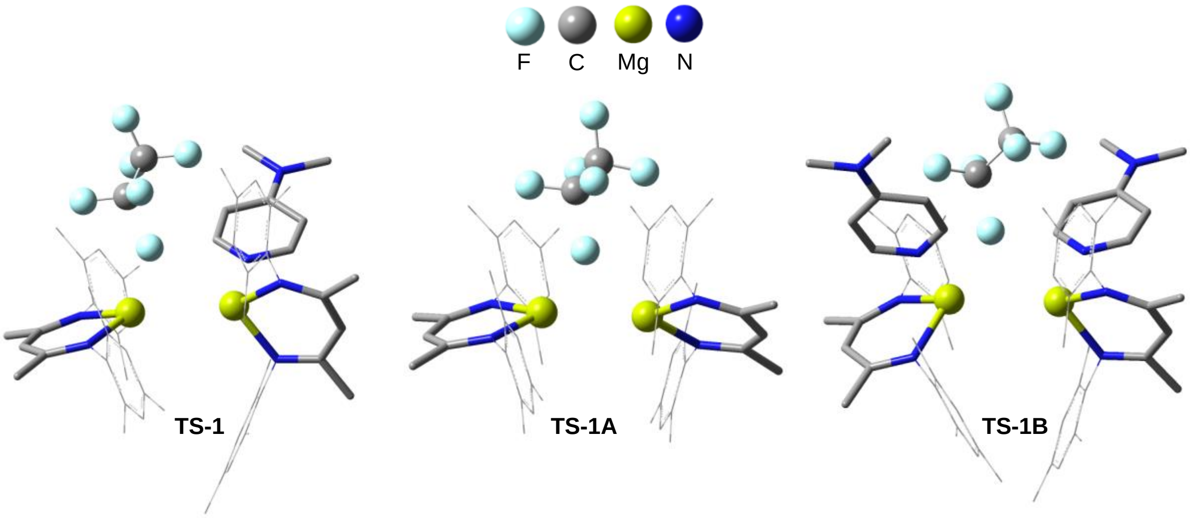

Figure S17: Calculated structures of **TS-1**, **TS-1A**, **TS-1B**.

Many other geometries of **TS-1** were considered where the orientation of the C_2F_6 fragment was varied, along with the position of the DMAP ligand, but all other variations were found to be much higher in energy than **TS-1**. A transition state where **1-(DMAP)** attacks the carbon atom of C_2F_6 in an S_N2-type fashion could not be located, despite many attempts and variations of orientation. We also explored the possibility of single electron transfer from the **1-(DMAP)** complex to C_2F_6 as an alternative pathway for C–F activation. Calculations revealed that electron transfer to form a triplet radical-anion radical-cation pair was prohibitively high in energy compared to **TS-1** ($\Delta G^{\ddagger}_{298K}$ = 45 kcal mol^{-1}) (Figure S18).

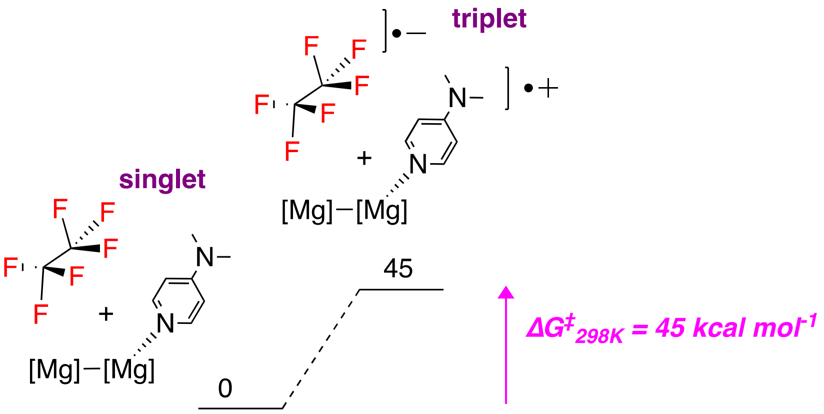

Figure S18: Calculated potential Energy Surface for single-electron transfer from **1-DMAP** to C_2F_6**.**

We could also support this result through experiment, where addition of the radical trapping reagent 9,10-dihydroanthracene to the optimised reaction for **1** + DMAP + PTFE led to no change, giving the same product **2** in 85 % yield (Scheme S12).[6]

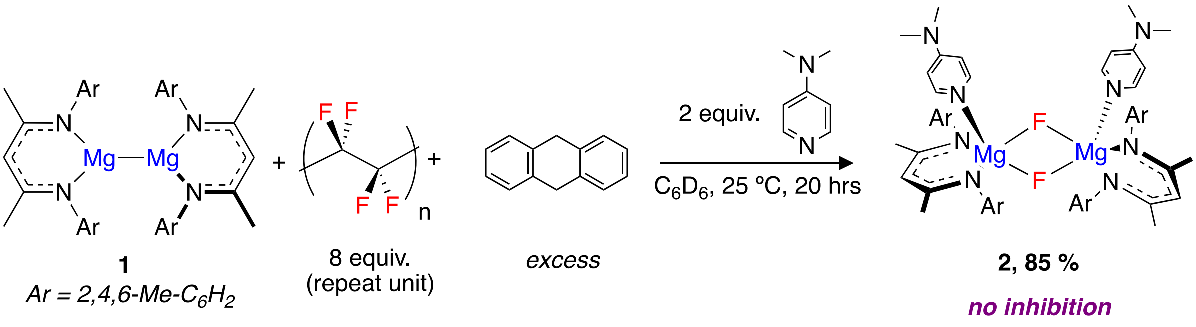

Scheme S12: Addition of a radical trapping reagent 9,10-dihydroanthracene to **1** + DMAP did not alter the outcome of the reaction

5.3. NBO Data for TS-1, TS-1A and TS-1B

A full NBO analysis was carried out and the relevant NPA charges are tabulated below.

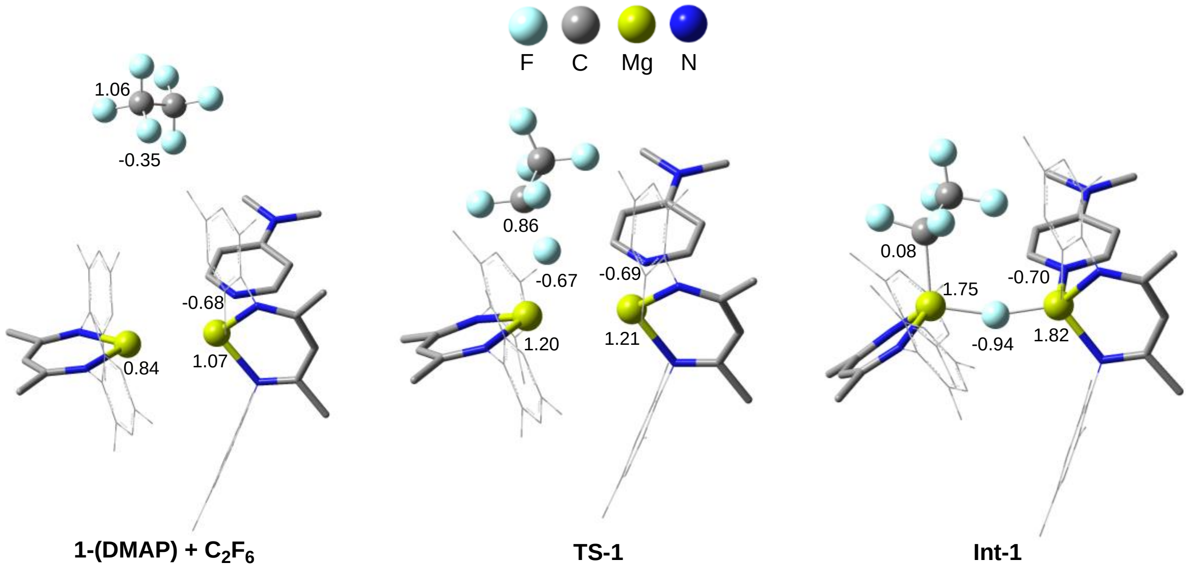

	1 + DMAP + C_2F_6	TS-1	Int-1
**Mg**	0.84	1.20	1.75
**Mg_{DMAP}**	1.07	1.21	1.82
**F**	-0.35	-0.67	-0.94
**C**	1.06	0.86	0.08
**N_{DMAP}**	-0.68	-0.69	-0.70

Table S3: NBO data for as **TS-1** is traversed.

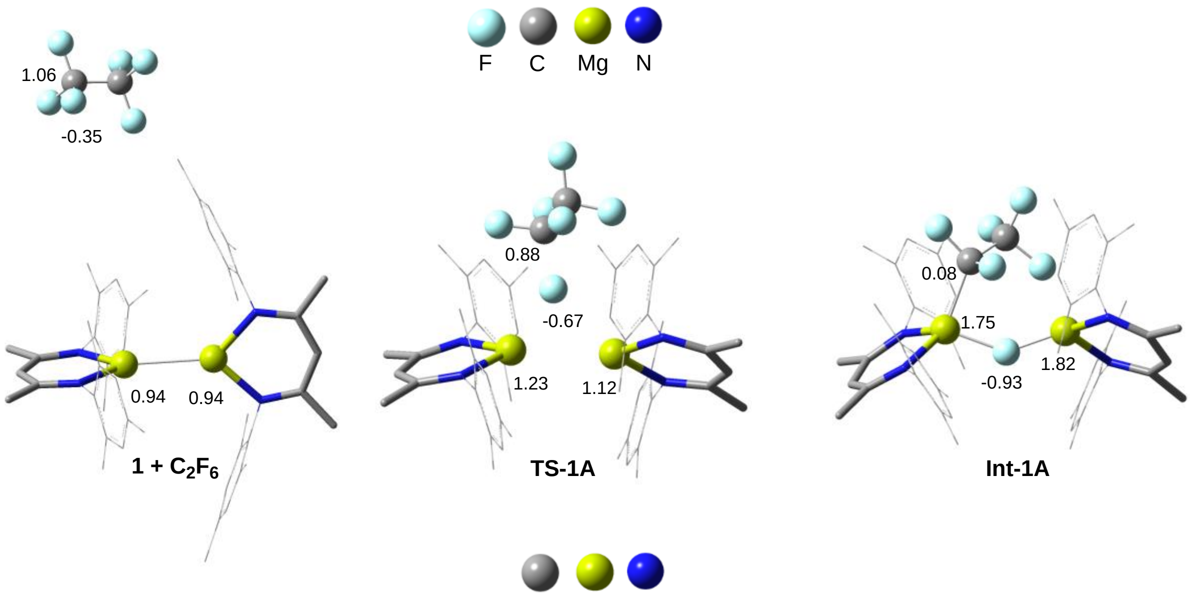

	1 + C_2F_6	TS-1A	Int-1A
**Mg**	0.94	1.23	1.75
**Mg**	0.94	1.12	1.82
**F**	-0.35	-0.67	-0.93
**C**	1.06	0.88	0.08

Table S4: NBO data for as **TS-1A** is traversed.

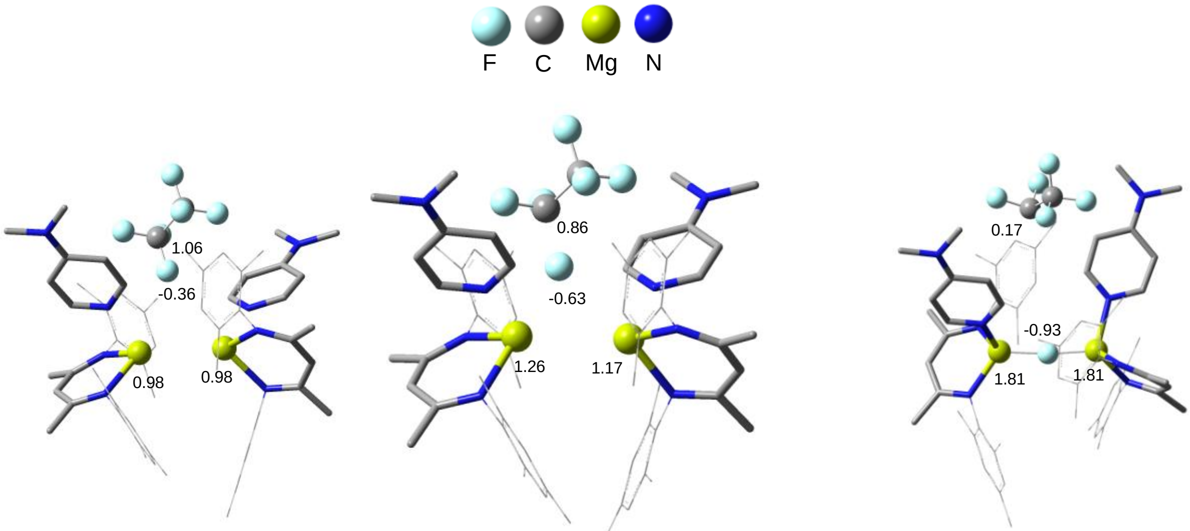

1-(DMAP)$_2$ + C_2F_6 **TS-1B** **Int-1**

	**1 + C_2F_6**	**TS-1B**	**Int-1B**
**Mg_{DMAP}**	0.98	1.17	1.81
**Mg_{DMAP}**	0.98	1.25	1.81
**F**	-0.36	-0.63	-0.93
**C**	1.06	0.86	0.17
**N_{DMAP}**	-0.66	-0.68	-0.72
**N_{DMAP}**	-0.66	-0.68	-0.72

Table S5: NBO data for as **TS-1B** is traversed.

5.4. Assessment of the Functional

An assessment of the computational methodology was carried out by a series of functional benchmarking calculations (Table S6). The functionals tested were the Minnesota hybrid-meta functional M06-2X,[17] the long-range corrected functional (with Grimme's D2 dispersion correction) ω-B97XD.[28,29] The same basis set and pseudopotential combination were maintained throughout. Single point solvent corrections (benzene, ε = 2.2706) were applied using the polarizable continuum model (PCM) to free energies.[20] Single point dispersion corrections using Grimme's D3 correction were applied to free energies in the cases of M06-2X and ω-B97X (noting that ω-B97X-D has Grimme's D2 dispersion correction built into the functional).[21] Consistent results were found across the different functionals. The chosen methodology, with B3PW91 as the functional, follows previous work in our group for C–F bond cleavage using Mg–Mg nucleophiles.[30]

It is noted that the functional benchmarking was carried out using the basis-set package BS4.

	ΔG‡ to **TS1**	ΔG‡ to **TS1A**	ΔG‡ to **TS1B**
B3PW91	31.7	36.7	45.0
M062X	45.8	48.5	50.6
wB97xD	43.0	49.3	50.2

Table S6: Relative free-energy barriers for the **TS-1**, **TS-1A** and **TS-1B** calculated for various density functionals. Free-energies in kcal mol^{-1}. BS3 was used for this functional assessment.

5.5. Assessment of Basis Sets

An assessment of the computational methodology was carried out by a series of basis set benchmarking calculations (Table S7). Calculated using B3PW91, with single point solvent (pcm, solvent=benzene) and dispersion (gd3bj) corrections.

	$\Delta G^{\ddagger}$ to **TS1**	$\Delta G^{\ddagger}$ to **TS1A**	$\Delta G^{\ddagger}$ to **TS1B**	$\Delta G^{\ddagger}$ to **TS2**
BS1	25.3	30.3	38.0	26.4
BS2	25.5	30.2	38.2	25.1
BS3	25.3	30.2	38.1	25.3
BS4	31.7	36.7	45.0	26.7

Table S7: Relative free-energy barriers for the **TS-1**, **TS-1A** and **TS-1B** calculated for various basis-sets. Free-energies in kcal mol^{-1}.

5.6. XYZ Coordinates

1_DMAP2.log

Mg	1.874662	8.522799	5.868280
N	-0.037223	8.413599	6.875384
C	-2.459378	8.020307	7.089589
H	-2.338358	7.367065	7.958692
H	-3.289282	7.651599	6.483808
H	-2.733664	9.006842	7.479659
Mg	4.292082	10.050731	6.891157
N	0.824446	8.576325	3.982930
C	-1.182296	8.115082	6.274419
N	2.225871	6.287980	5.839946
C	-1.331210	7.909960	4.885557
H	-2.329197	7.625404	4.571697
N	2.825145	2.128832	5.837512
C	-0.443986	8.219600	3.829529
N	6.347639	9.603291	6.383860
C	-1.047248	8.166928	2.437305
H	-0.422988	7.582890	1.754483
H	-1.116109	9.172790	2.009178
H	-2.049712	7.735120	2.456834
N	4.794003	12.150684	6.865453
C	-0.074430	8.757612	8.255329
C	0.084437	7.778730	9.256126
C	0.045691	8.167265	10.600701
H	0.144143	7.399049	11.367054
C	-0.128465	9.496511	10.985622
C	-0.262002	10.453114	9.975703
H	-0.395931	11.499337	10.246107
C	-0.237916	10.111637	8.622737
C	0.291806	6.331500	8.899908
H	1.231818	6.191446	8.356875
C	-0.375818	11.170492	7.565290
H	-1.220573	10.974575	6.895894
C	1.519810	9.062251	2.836991
C	1.434990	10.433567	2.514803
C	2.124242	10.916425	1.401042
H	2.044744	11.974751	1.157035
C	2.904523	10.085686	0.593883
C	2.977867	8.735377	0.934450
H	3.572394	8.063143	0.317271
C	2.307023	8.207069	2.042119
C	0.614799	11.364497	3.364841
H	-0.431867	11.047816	3.433072
C	2.432168	6.742248	2.361904
H	1.458110	6.274425	2.537514
C	1.230298	5.432731	5.554913
H	0.266257	5.884493	5.329928
C	1.375030	4.057330	5.535049
H	0.514633	3.445727	5.292367
C	2.633107	3.481982	5.829514
C	3.679250	4.389235	6.112128
H	4.686254	4.054142	6.325818
C	3.425320	5.749943	6.104497
H	4.225379	6.458262	6.312434
C	1.739235	1.239608	5.476191
H	0.880302	1.360616	6.147937
H	2.081182	0.207265	5.555870
H	1.397029	1.405625	4.445694
C	4.142695	1.585860	6.101188
H	4.874124	1.889657	5.340190
H	4.086897	0.496835	6.101606
H	4.516443	1.902908	7.082505
C	6.269207	14.114934	7.024357
H	5.948433	14.618903	6.106240
H	7.325380	14.339035	7.185607
H	5.685398	14.555318	7.838158
C	6.033921	12.618615	6.920923
C	7.202284	11.825763	6.850811
H	8.131733	12.378279	6.930148
C	7.348005	10.469466	6.487541
C	8.763315	10.029214	6.159619
H	9.034244	9.110546	6.688226
H	9.487624	10.806851	6.409297
H	8.854188	9.809185	5.090085
C	3.733734	13.095464	6.739027
C	3.350787	13.525944	5.450907
C	2.305903	14.442458	5.320591
H	2.024518	14.774125	4.322244
C	1.618766	14.946156	6.427187
C	2.010727	14.502218	7.689799
H	1.497294	14.885061	8.570778
C	3.049261	13.582771	7.869053
C	4.059367	12.999787	4.233274
H	3.952308	11.911353	4.161204
C	3.418967	13.135495	9.257256
H	4.497039	13.198080	9.436960
C	6.627609	8.319214	5.839220
C	6.974802	7.234947	6.669228
C	7.235144	5.988259	6.087825
H	7.527539	5.162875	6.736223
C	7.146707	5.776839	4.711959
C	6.781603	6.861559	3.910245
H	6.702552	6.725685	2.832772
C	6.520563	8.124279	4.444421
C	7.071754	7.401169	8.161576
H	6.099861	7.662775	8.591800
C	6.124222	9.264649	3.549270
H	5.124016	9.629325	3.812386
H	6.104980	8.953884	2.501636
H	6.805499	10.117236	3.644829
H	7.419415	6.477935	8.634600
H	7.759565	8.205851	8.442653
C	7.419454	4.424595	4.105650
H	6.492284	3.940085	3.774077
H	8.069804	4.501758	3.227532
H	7.906106	3.755638	4.822269
H	3.649742	13.441680	3.321021
H	5.134914	13.207580	4.259517
H	3.135809	12.091033	9.422290
H	2.910709	13.746181	10.009382
C	0.478783	15.916757	6.258197
H	-0.455170	15.397557	6.008742
H	0.299401	16.487116	7.174786
H	0.675256	16.630407	5.451207
H	0.636914	12.381324	2.963627
H	0.999925	11.395911	4.390461
H	2.926286	6.208014	1.544971
H	3.020774	6.587295	3.271767
C	3.658214	10.636477	-0.588800
H	3.076951	11.401049	-1.114869
H	4.601083	11.104857	-0.279494
H	3.906050	9.849271	-1.307465
H	-0.511338	12.158379	8.012612
H	0.523542	11.203745	6.937983
H	0.319187	5.709159	9.799313
H	-0.500663	5.949648	8.247498
C	-0.159452	9.896773	12.438012

H	-0.241247	9.023033	13.092007
H	0.748862	10.442846	12.722468
H	-1.007634	10.555286	12.655266
C	3.659509	9.183798	9.890040
C	3.795731	8.996117	11.254685
C	4.947472	9.481139	11.914731
C	5.890090	10.155493	11.103983
C	5.654984	10.287771	9.747402
N	4.565009	9.810277	9.124662
H	2.772755	8.822313	9.372282
H	3.001983	8.487190	11.786592
H	6.798696	10.574058	11.519315
H	6.374556	10.804345	9.115395
N	5.138977	9.311264	13.257164
C	6.307957	9.876414	13.900922
H	6.338862	10.970347	13.808053
H	6.285938	9.629088	14.962673
H	7.236042	9.468846	13.480688
C	4.130887	8.640687	14.053629
H	3.946854	7.621730	13.691177
H	4.478426	8.570548	15.084821
H	3.176239	9.183612	14.052024

1_DMAP.log

Mg	0.253912	-1.332381	0.820862
N	1.163596	-2.761061	-0.464907
N	-1.300501	-2.715098	1.276215
C	1.700034	-5.025793	-1.261266
C	0.900363	-4.060896	-0.406577
C	-0.116326	-4.639652	0.385869
C	-1.193807	-4.020215	1.060112
C	-2.301686	-4.948810	1.520042
C	2.069962	-2.291189	-1.459442
C	3.428747	-2.074613	-1.158605
C	4.273721	-1.573161	-2.153297
C	3.815458	-1.269978	-3.435377
C	2.463915	-1.489352	-3.709899
C	1.582868	-1.989449	-2.748783
C	3.978664	-2.376399	0.209085
C	4.739268	-0.701254	-4.480867
C	0.129935	-2.190508	-3.082592
C	-2.533574	-2.209277	1.784748
C	-2.724460	-2.003634	3.165037
C	-3.939923	-1.475925	3.610873
C	-4.970323	-1.136856	2.733878
C	-4.756472	-1.345260	1.369934
C	-3.559488	-1.869816	0.878444
C	-1.649632	-2.345724	4.161165
C	-6.259521	-0.542081	3.238032
C	-3.361331	-2.058896	-0.599989
H	2.775450	-4.856565	-1.151746
H	1.470116	-4.880743	-2.322403
H	1.476156	-6.062620	-1.003361
H	-0.170484	-5.721428	0.338078
H	-2.015952	-5.995610	1.401160
H	-3.212797	-4.774995	0.937227
H	-2.565037	-4.768869	2.566562
H	5.324933	-1.419058	-1.914175
H	2.079202	-1.265899	-4.703787
H	3.726380	-3.389682	0.539286
H	3.567511	-1.694135	0.960108
H	5.067841	-2.275940	0.220291
H	5.780617	-0.976959	-4.288025
H	4.477427	-1.055220	-5.483201
H	4.691401	0.394827	-4.502691
H	-0.067373	-1.944989	-4.129603
H	-0.197626	-3.220963	-2.907518
H	-0.505851	-1.551420	-2.459206
H	-4.082940	-1.330585	4.680686
H	-5.544247	-1.093304	0.661828
H	-2.032362	-2.274946	5.183488
H	-1.256987	-3.356937	4.012948
H	-0.796412	-1.665572	4.072355
H	-6.222442	0.554670	3.233537
H	-6.470558	-0.854740	4.265430
H	-7.109024	-0.838942	2.614671
H	-4.258317	-1.772931	-1.154434
H	-3.113848	-3.095108	-0.855382
H	-2.533016	-1.438414	-0.961048
Mg	-0.014760	1.555637	0.177193
N	-1.119448	2.759689	-1.155811
N	0.980114	3.276035	0.894741
C	-1.868496	4.804499	-2.302915
C	-1.001255	4.077064	-1.294546
C	-0.108661	4.880476	-0.558434
C	0.803615	4.518673	0.447688
C	1.625560	5.648702	1.036018
C	-2.067136	2.082858	-1.977785
C	-3.385108	1.892751	-1.521224
C	-4.296579	1.228516	-2.346569
C	-3.936086	0.730018	-3.599370
C	-2.615656	0.909551	-4.018002
C	-1.671865	1.572347	-3.230338
C	-3.804711	2.393394	-0.165403
C	-4.928913	-0.005332	-4.461895
C	-0.256597	1.742981	-3.713138
C	1.928337	3.067776	1.933372
C	1.527127	3.151030	3.281169
C	2.480634	2.953586	4.286106
C	3.814219	2.662106	3.994014
C	4.176707	2.537172	2.649771
C	3.260549	2.729909	1.614278
C	0.093871	3.445870	3.633535
C	4.841183	2.509951	5.086676
C	3.684584	2.578936	0.178646
H	-2.930567	4.658757	-2.080787
H	-1.708631	4.413394	-3.312761
H	-1.657779	5.875210	-2.306708
H	-0.131156	5.936074	-0.802298
H	1.380952	6.602365	0.565382
H	2.696429	5.460745	0.905942
H	1.456503	5.739530	2.113902
H	-5.319407	1.098720	-1.996416
H	-2.308630	0.526677	-4.990070
H	-3.609176	3.464043	-0.041213
H	-3.253494	1.876915	0.630108
H	-4.870900	2.218975	0.002331
H	-5.958376	0.256837	-4.199689
H	-4.783973	0.221487	-5.523168
H	-4.829376	-1.092096	-4.347998
H	-0.135145	1.329639	-4.718162
H	0.045916	2.795607	-3.740035
H	0.452857	1.230769	-3.051846
H	2.168830	3.040942	5.325977
H	5.206331	2.289485	2.396353
H	-0.236931	4.413067	3.239242
H	-0.578651	2.691814	3.206436
H	-0.049570	3.454835	4.717639
H	5.480610	1.635724	4.920828
H	4.370099	2.406509	6.069168
H	5.502939	3.383495	5.135493
H	4.753323	2.359204	0.107016
H	3.480819	3.482196	-0.406483
H	3.138918	1.760736	-0.308059
C	1.713037	-2.975904	3.028176

C	1.966141	-0.739670	3.434258
C	2.399547	-3.320378	4.177081
H	1.314000	-3.751612	2.377742
C	2.662325	-0.974985	4.606310
H	1.778981	0.281134	3.107041
H	2.531072	-4.369707	4.410566
H	3.008033	-0.123860	5.178439
N	1.483879	-1.709049	2.641353
C	2.905196	-2.304374	5.021654
N	3.586660	-2.591572	6.169047
C	4.034244	-1.517817	7.034373
H	3.193759	-0.924370	7.418239
H	4.564660	-1.943366	7.886601
H	4.723085	-0.842310	6.512826
C	3.779172	-3.970317	6.574116
H	4.364819	-3.993981	7.493447
H	2.825168	-4.479733	6.765604
H	4.327612	-4.538877	5.813065

1.log

Mg	0.068587	-1.436586	0.021899
N	1.182649	-2.842896	-1.015437
N	-0.916770	-2.916305	1.089603
C	2.000322	-5.070703	-1.653259
C	1.108793	-4.159583	-0.835688
C	0.244022	-4.788339	0.081178
C	-0.693839	-4.222763	0.966742
C	-1.483036	-5.193410	1.820260
C	2.088112	-2.316671	-1.981604
C	3.386867	-1.935847	-1.591507
C	4.240705	-1.373988	-2.543861
C	3.842124	-1.165522	-3.865730
C	2.544006	-1.536294	-4.222521
C	1.655554	-2.102145	-3.304997
C	3.843808	-2.119780	-0.169046
C	4.771554	-0.529830	-4.866758
C	0.256263	-2.468300	-3.722147
C	-1.895853	-2.457503	2.017717
C	-1.515066	-2.125714	3.332684
C	-2.481913	-1.631139	4.211497
C	-3.809745	-1.442168	3.822990
C	-4.157344	-1.762352	2.509051
C	-3.225140	-2.260333	1.595436
C	-0.087414	-2.289954	3.780840
C	-4.827245	-0.879232	4.780995
C	-3.633049	-2.571249	0.180143
H	3.056016	-4.830998	-1.489644
H	1.816308	-4.938841	-2.724386
H	1.837669	-6.119218	-1.398791
H	0.307008	-5.869866	0.106175
H	-1.193268	-6.226013	1.619384
H	-2.557278	-5.090199	1.635657
H	-1.331790	-4.988642	2.885050
H	5.247191	-1.090356	-2.240455
H	2.208492	-1.380412	-5.246561
H	3.734046	-3.155299	0.170437
H	3.254638	-1.500551	0.519342
H	4.893230	-1.834378	-0.056185
H	5.818618	-0.752009	-4.638940
H	4.566273	-0.879941	-5.883096
H	4.665179	0.562283	-4.872085
H	0.097085	-2.255895	-4.782766
H	0.037547	-3.528188	-3.551782
H	-0.489402	-1.901312	-3.151472
H	-2.185484	-1.385933	5.230084
H	-5.185893	-1.620638	2.181141
H	0.280338	-3.310083	3.626948
H	0.582695	-1.627773	3.218078
H	0.019140	-2.046801	4.841502
H	-4.859198	0.216416	4.730285
H	-4.595365	-1.148724	5.816003
H	-5.834377	-1.243253	4.555628
H	-4.703772	-2.402712	0.036624
H	-3.412117	-3.607930	-0.096163
H	-3.095983	-1.936589	-0.535754
Mg	-0.068631	1.436578	0.021474
N	-1.182815	2.842594	-1.016118
N	0.916853	2.916595	1.088658
C	-2.000603	5.070216	-1.654435
C	-1.108961	4.159329	-0.836726
C	-0.244096	4.788342	0.079874
C	0.693886	4.223017	0.965471
C	1.483179	5.193908	1.818623
C	-2.088359	2.316095	-1.982059
C	-3.387064	1.935330	-1.591738
C	-4.240977	1.373201	-2.543867
C	-3.842516	1.164405	-3.865720
C	-2.544444	1.535126	-4.222731
C	-1.655923	2.101243	-3.305438
C	-3.843872	2.119599	-0.169278
C	-4.772022	0.528423	-4.866494
C	-0.256683	2.467337	-3.722814
C	1.896074	2.458070	2.016766
C	1.515478	2.126641	3.331880
C	2.482463	1.632348	4.210699
C	3.810247	1.443307	3.822064
C	4.157653	1.763125	2.507985
C	3.225307	2.260819	1.594358
C	0.087883	2.290953	3.780190
C	4.827896	0.880679	4.780091
C	3.633012	2.571339	0.178920
H	-3.056273	4.830532	-1.490633
H	-1.816712	4.938074	-2.725549
H	-1.837941	6.118802	-1.400263
H	-0.307098	5.869874	0.104593
H	1.193358	6.226451	1.617514
H	2.557398	5.090674	1.633895
H	1.332088	4.989417	2.883487
H	-5.247425	1.089616	-2.240291
H	-2.209023	1.378988	-5.246762
H	-3.734023	3.155184	0.169972
H	-3.254678	1.500482	0.519191
H	-4.893299	1.834278	-0.056264
H	-5.819072	0.750618	-4.638626
H	-4.566857	0.878285	-5.882941
H	-4.665604	-0.563686	-4.871555
H	-0.097571	2.254588	-4.783374
H	-0.038014	3.527295	-3.552819
H	0.489055	1.900585	-3.152001
H	2.186182	1.387425	5.229398
H	5.186161	1.621348	2.179973
H	-0.279969	3.310995	3.625959
H	-0.582262	1.628500	3.217788
H	-0.018492	2.048201	4.840962
H	4.859800	-0.214988	4.729771
H	4.596208	1.150552	5.815044
H	5.835000	1.244584	4.554415
H	4.703725	2.402816	0.035308
H	3.411988	3.607925	-0.097666
H	3.095886	1.936435	-0.536717

2.log

Mg	16.922598	14.507939	14.521652
Mg	14.986237	14.499328	12.056137
F	15.962071	13.270149	13.287297
F	15.947349	15.704864	13.289952
N	17.362915	16.004681	16.002209
N	14.518729	15.997250	10.584918
N	16.583703	12.923327	16.043143
N	18.980784	14.226120	14.151168
N	15.355491	12.930091	10.526236
N	12.933197	14.178914	12.424518
N	16.209078	9.731552	18.750990
N	15.785753	9.757482	7.804187
C	16.331374	10.768421	17.869643
C	16.333551	18.194688	15.645037
C	15.645403	10.788138	8.690103
C	16.376045	16.974485	16.354247
C	15.487326	16.987709	10.239803
C	12.540178	13.525947	13.626770
C	19.928523	14.618353	14.996996
C	17.436011	12.764136	17.068667
H	18.227347	13.506262	17.148315
C	15.506362	18.203929	10.956746
C	17.407404	18.970821	9.625070
C	19.710253	15.397376	16.152618
H	20.599672	15.596612	16.739579
C	14.507511	12.762046	9.498525
H	13.704363	13.491612	9.421141
C	16.461432	19.168631	10.633988
H	16.463298	20.105173	11.189617
C	15.607939	12.008723	15.921572
H	14.942157	12.142099	15.074902
C	15.360522	19.139021	15.974301
H	15.340597	20.078823	15.424564
C	16.430888	16.765094	9.219454
C	11.979018	14.557330	11.579599
C	17.361927	11.730111	17.983859
H	18.101557	11.678644	18.773345
C	18.564316	16.128224	16.547782
C	13.315720	16.101476	10.038855
C	19.384582	13.587207	12.944960
C	14.599731	11.733697	8.578598
H	13.862272	11.673999	7.787668
C	15.437956	10.943379	16.788810
H	14.614792	10.262655	16.612889
C	16.534144	10.972360	9.773254
H	17.367891	10.304175	9.947380
C	15.437589	16.727654	17.373757
C	14.419115	18.916970	16.982459
C	17.368945	17.762981	8.930831
H	18.085385	17.586901	8.129595
C	12.453843	12.121518	13.691329
C	12.269710	14.299978	14.775117
C	14.480960	17.705781	17.669105
H	13.768542	17.511171	18.469637
C	12.183537	15.346628	10.428451
H	11.291075	15.532172	9.841614
C	16.345659	12.030756	10.645138
H	17.007877	12.170685	11.493526
C	17.316213	18.469073	14.540660
H	18.354609	18.387614	14.880574
H	17.172194	19.473482	14.133139
H	17.182609	17.743434	13.732346
C	14.518318	18.452596	12.062420
H	13.481755	18.352413	11.721941
H	14.642395	19.457341	12.475641
H	14.666211	17.725307	12.866757
C	18.789660	17.140227	17.656261
H	18.724138	18.160613	17.263499
H	19.773894	17.013971	18.111311
H	18.024670	17.058498	18.433976
C	18.439978	20.022624	9.312688
H	17.998391	21.024801	9.289460
H	18.913925	19.842433	8.343048
H	19.235080	20.039636	10.068454
C	19.498060	12.185077	12.872847
C	21.375366	14.257943	14.715917
H	21.495241	13.185144	14.537423
H	22.023859	14.552215	15.542924
H	21.725935	14.763563	13.809477
C	15.159821	8.748038	18.571601
H	14.164637	9.208386	18.610615
H	15.219441	8.011320	19.373272
H	15.256130	8.217582	17.614875
C	17.165041	9.578742	19.829494
H	18.184584	9.417187	19.454428
H	16.887523	8.714343	20.433402
H	17.174876	10.457793	20.486151
C	19.638059	14.372437	11.800323
C	13.072223	17.115901	8.936455
H	13.117737	18.134852	9.335727
H	12.091060	16.973681	8.479478
H	13.839472	17.053766	8.159156
C	16.441381	15.487284	8.424184
H	16.776131	14.640310	9.031200
H	17.114452	15.572486	7.565881
H	15.445207	15.228511	8.051108
C	12.060752	11.520569	14.892163
H	11.971057	10.435282	14.926273
C	10.539076	14.167503	11.856836
H	10.439712	13.091536	12.028789
H	9.885940	14.454144	11.030804
H	10.177851	14.660836	12.765846
C	11.892089	13.655447	15.955337
H	11.676327	14.261683	16.833657
C	15.451860	15.445044	18.161196
H	15.129302	14.596119	17.550287
H	14.780591	15.513510	19.022409
H	16.453700	15.201139	18.528952
C	12.781757	11.273281	12.492782
H	12.201183	11.563425	11.610670
H	12.583861	10.216831	12.697309
H	13.836827	11.380171	12.221304
C	12.377337	15.799887	14.723750
H	12.166284	16.241332	15.700786
H	11.681742	16.231968	13.995183
H	13.380480	16.116073	14.420417
C	11.773624	12.265795	16.036606
C	13.367096	19.947125	17.302013
H	13.790122	20.957067	17.333302
H	12.895873	19.750802	18.269843
H	12.572378	19.955305	16.545704
C	14.833744	9.594031	6.723731
H	13.816517	9.413930	7.096652
H	15.126262	8.737224	6.116092
H	14.810278	10.475913	6.071248
C	16.849828	8.789593	7.981325
H	17.837766	9.265595	7.946572
H	16.803385	8.055802	7.176109
H	16.759823	8.253244	8.935385
C	19.189296	11.324439	14.067656
H	19.768487	11.619298	14.949148
H	19.404400	10.272728	13.856214
H	18.133720	11.411710	14.344080
C	19.501607	15.869670	11.860038
H	18.492733	16.164710	12.165701
H	19.703545	16.320535	10.885383

H	20.189240	16.310911	12.590660
C	20.026007	13.741556	10.616087
H	20.228506	14.356478	9.740656
C	19.900492	11.598185	11.668150
H	20.011113	10.515034	11.628122
C	20.171077	12.354884	10.527211
C	11.372874	11.597701	17.326315
H	10.571081	12.146831	17.831079
H	12.216101	11.545888	18.026925
H	11.021401	10.575340	17.155191
C	20.582062	11.701495	9.233206
H	21.371648	12.269005	8.729538
H	19.738378	11.636615	8.534232
H	20.954099	10.685427	9.398238

C2F6.log

C	-3.699089	1.516876	-0.008421
C	-3.178874	2.252558	1.265826
F	-3.260310	2.137391	-1.099215
F	-5.028492	1.512266	-0.016434
F	-3.260335	0.261954	-0.016435
F	-3.617628	3.507480	1.273840
F	-1.849471	2.257169	1.273838
F	-3.617653	1.632044	2.356620

DMAP.log

C	-3.579211	1.210766	0.739671
C	-2.199662	1.360313	0.772561
C	-1.589338	2.330457	-0.052013
C	-2.464646	3.099516	-0.849465
C	-3.829507	2.853515	-0.792302
N	-4.410439	1.929282	-0.021248
H	-4.046907	0.459837	1.374917
H	-1.616916	0.729215	1.432869
H	-2.096929	3.879605	-1.505322
H	-4.501991	3.446625	-1.410490
N	-0.229358	2.513276	-0.078055
C	0.335330	3.605554	-0.842876
H	0.007722	4.589423	-0.476289
H	1.423297	3.563260	-0.776333
H	0.065843	3.527629	-1.902745
C	0.611811	1.789555	0.852311
H	0.495535	0.706154	0.730940
H	1.656371	2.032465	0.652870
H	0.395152	2.043298	1.900351

Int_1.log

Mg	0.279870	-1.693839	-0.253721
N	1.353075	-2.784613	-1.655634
N	-1.221801	-3.112045	-0.083629
C	2.174653	-4.932678	-2.535168
C	1.209643	-4.110893	-1.703803
C	0.187080	-4.834629	-1.060349
C	-1.000057	-4.371129	-0.443887
C	-2.084899	-5.410316	-0.250010
C	2.266473	-2.182798	-2.574243
C	3.579375	-1.857419	-2.185780
C	4.429879	-1.251393	-3.115831
C	4.017607	-0.946607	-4.413115
C	2.711377	-1.282574	-4.775907
C	1.829198	-1.896103	-3.884625
C	4.081093	-2.164912	-0.800413
C	4.941299	-0.260485	-5.385066
C	0.437042	-2.262363	-4.324878
C	-2.522599	-2.773287	0.414680
C	-2.823698	-2.904105	1.783656
C	-4.097031	-2.542863	2.229234
C	-5.073413	-2.044679	1.365749
C	-4.748520	-1.926315	0.014236
C	-3.490170	-2.277831	-0.478843
C	-1.803350	-3.412285	2.767139
C	-6.419567	-1.611832	1.882882
C	-3.180346	-2.118848	-1.942913
H	3.212927	-4.670906	-2.310711
H	2.031016	-4.740100	-3.603494
H	2.032723	-6.000362	-2.360023
H	0.230086	-5.907270	-1.208920
H	-1.717198	-6.411919	-0.478433
H	-2.927852	-5.194258	-0.915804
H	-2.485737	-5.399119	0.766619
H	5.449252	-1.016415	-2.813701
H	2.367106	-1.066310	-5.785830
H	3.857780	-3.194662	-0.503052
H	3.615680	-1.516418	-0.050514
H	5.163482	-2.020353	-0.739727
H	4.778957	0.824482	-5.388543
H	5.991201	-0.431806	-5.129251
H	4.781242	-0.613985	-6.408563
H	0.255547	-1.933733	-5.351558
H	0.264902	-3.344079	-4.285913
H	-0.321946	-1.806899	-3.681203
H	-4.326095	-2.644678	3.288462
H	-5.491133	-1.540184	-0.681210
H	-2.256499	-3.554679	3.752013
H	-1.362465	-4.366108	2.457371
H	-0.979318	-2.701438	2.881181
H	-6.400940	-0.556016	2.178641
H	-6.718478	-2.191855	2.761585
H	-7.197568	-1.724220	1.121421
H	-4.077340	-1.841787	-2.501373
H	-2.770674	-3.035270	-2.381069
H	-2.443639	-1.324895	-2.112060
Mg	-0.544884	2.021072	-0.264452
N	-1.314574	2.962140	-1.959902
N	1.055842	3.355691	-0.104104
C	-1.551584	4.818227	-3.557193
C	-0.875086	4.147899	-2.377571
C	0.213897	4.850114	-1.824030
C	1.132798	4.470819	-0.828592
C	2.302501	5.412947	-0.622432
C	-2.336271	2.328927	-2.732683
C	-3.698602	2.515721	-2.427294
C	-4.655544	1.852317	-3.201712
C	-4.308838	1.018342	-4.265621
C	-2.951431	0.852919	-4.548931
C	-1.958940	1.494354	-3.804133
C	-4.133364	3.419929	-1.305465
C	-5.366106	0.353317	-5.108900
C	-0.506130	1.318733	-4.157518
C	2.107470	3.081514	0.817350
C	2.053664	3.565723	2.139658
C	3.092047	3.236104	3.019038
C	4.182272	2.458649	2.627418
C	4.211534	1.989781	1.310806
C	3.195706	2.285281	0.399732
C	0.936071	4.463136	2.597902
C	5.315575	2.166283	3.577098
C	3.272501	1.787708	-1.018021
H	-2.625966	4.930636	-3.383696
H	-1.446304	4.212952	-4.463574

H	-1.123569	5.803312	-3.748655
H	0.417723	5.807796	-2.287634
H	2.213843	6.295900	-1.256993
H	3.247040	4.911485	-0.859455
H	2.376353	5.738638	0.418902
H	-5.707777	2.003023	-2.965865
H	-2.653064	0.213682	-5.378498
H	-3.603442	4.377274	-1.321738
H	-3.937585	2.966692	-0.329926
H	-5.206147	3.623188	-1.369779
H	-5.010194	-0.593500	-5.526983
H	-6.271156	0.147355	-4.529123
H	-5.660737	0.989556	-5.953202
H	-0.393486	0.663355	-5.024745
H	-0.026238	2.274790	-4.395126
H	0.065081	0.887483	-3.328677
H	3.047572	3.616272	4.038517
H	5.053285	1.384739	0.977348
H	-0.036094	4.133292	2.227923
H	0.891557	4.502410	3.689382
H	1.079323	5.490270	2.238549
H	5.674648	1.136570	3.472273
H	5.016374	2.320297	4.618602
H	6.173482	2.823483	3.387496
H	4.174817	1.192872	-1.178244
H	3.282212	2.615251	-1.736259
H	2.408592	1.167580	-1.277860
C	1.737531	-3.068990	1.977373
C	1.795001	-0.805739	2.332380
C	2.406336	-3.328255	3.156083
H	1.430423	-3.891680	1.334506
C	2.464101	-0.956898	3.530203
H	1.547360	0.190150	1.980733
H	2.612590	-4.357312	3.421849
H	2.720211	-0.065672	4.087410
N	1.416837	-1.832780	1.549905
C	2.790782	-2.252719	3.994362
N	3.435338	-2.454377	5.175687
C	3.746546	-1.325478	6.034657
H	2.840356	-0.789481	6.343898
H	4.248828	-1.688271	6.931410
H	4.416876	-0.615489	5.536178
C	3.721338	-3.802991	5.628315
H	4.262938	-3.754681	6.572958
H	2.803713	-4.383398	5.791542
H	4.350249	-4.341276	4.908999
C	-1.926740	1.910296	1.494699
C	-1.589545	1.174977	2.797271
F	0.006317	0.167200	-0.496050
F	-1.298775	-0.117472	2.540669
F	-2.564123	1.178553	3.721531
F	-0.497737	1.710367	3.381309
F	-2.305501	3.191699	1.915065
F	-3.124730	1.313821	1.083615

Int_2.log

Mg	0.321610	-1.317111	-0.383087
N	1.329948	-2.795045	-1.559571
N	-1.301978	-2.645162	-0.125203
C	1.915074	-5.102627	-2.194452
C	1.065550	-4.089434	-1.451577
C	-0.004011	-4.621813	-0.694878
C	-1.142484	-3.965790	-0.180919
C	-2.253795	-4.875501	0.308508
C	2.306135	-2.392896	-2.520091
C	3.646471	-2.181648	-2.148765
C	4.562670	-1.759690	-3.117487
C	4.191930	-1.531249	-4.441918
C	2.855367	-1.744704	-4.786311
C	1.907163	-2.165737	-3.853087
C	4.108012	-2.404822	-0.733990
C	5.190101	-1.049312	-5.462446
C	0.472771	-2.353788	-4.265102
C	-2.603353	-2.158504	0.204560
C	-2.918400	-1.769572	1.521163
C	-4.202919	-1.299625	1.801701
C	-5.186786	-1.191148	0.816768
C	-4.847899	-1.569987	-0.482199
C	-3.575131	-2.046750	-0.808872
C	-1.891770	-1.850927	2.616257
C	-6.559581	-0.664652	1.146695
C	-3.254320	-2.431717	-2.228114
H	2.982814	-4.919633	-2.042476
H	1.739411	-5.030580	-3.273346
H	1.679258	-6.121049	-1.879778
H	-0.039199	-5.704379	-0.647854
H	-1.925625	-5.916186	0.336767
H	-3.129078	-4.810468	-0.346150
H	-2.592565	-4.582237	1.306864
H	5.600249	-1.609680	-2.822282
H	2.536930	-1.576909	-5.814213
H	3.818473	-3.391528	-0.356773
H	3.668187	-1.670257	-0.051899
H	5.196526	-2.321465	-0.663635
H	5.025681	-1.518351	-6.438259
H	5.118651	0.035630	-5.610550
H	6.216604	-1.268719	-5.153165
H	0.353243	-2.199319	-5.341230
H	0.096128	-3.353047	-4.022138
H	-0.174637	-1.639433	-3.745731
H	-4.441441	-1.014669	2.825633
H	-5.594973	-1.497191	-1.270755
H	-2.353697	-1.681771	3.593685
H	-1.390173	-2.823865	2.640291
H	-1.116170	-1.093542	2.463266
H	-6.925945	-1.066340	2.097502
H	-7.285305	-0.926114	0.370663
H	-6.558701	0.428663	1.239577
H	-4.128221	-2.303697	-2.871664
H	-2.918930	-3.471345	-2.311173
H	-2.448102	-1.811620	-2.633521
Mg	-0.018208	1.660710	-0.915834
N	-1.193387	2.738177	-2.201180
N	1.001652	3.297929	-0.216964
C	-1.974830	4.766208	-3.346523
C	-1.084407	4.056154	-2.348433
C	-0.178237	4.871560	-1.639645
C	0.785031	4.532358	-0.672592
C	1.626183	5.674662	-0.141737
C	-2.128150	2.039655	-3.024226
C	-3.441184	1.823531	-2.568359
C	-4.335041	1.136820	-3.395279
C	-3.960025	0.646598	-4.646641
C	-2.639068	0.843157	-5.057918
C	-1.711729	1.527849	-4.269842
C	-3.874491	2.311311	-1.211591
C	-4.949871	-0.056710	-5.538742
C	-0.292448	1.709312	-4.739306
C	2.003170	3.106703	0.776945
C	1.671361	3.225691	2.140375
C	2.673730	3.033605	3.098000
C	3.984087	2.709001	2.742778
C	4.275710	2.554786	1.384360
C	3.311399	2.744567	0.392694

C	0.260975	3.543657	2.560870
C	5.060702	2.543660	3.784200
C	3.660566	2.566613	-1.061006
H	-3.031403	4.594697	-3.117701
H	-1.811338	4.379991	-4.357774
H	-1.787711	5.840976	-3.349502
H	-0.221594	5.924744	-1.890153
H	1.349224	6.620273	-0.609862
H	2.689450	5.491180	-0.328678
H	1.515781	5.777328	0.942431
H	-5.353554	0.980526	-3.044970
H	-2.317709	0.454387	-6.022827
H	-3.344528	1.778889	-0.413316
H	-4.944643	2.145274	-1.063767
H	-3.671609	3.378742	-1.073284
H	-5.314400	0.608793	-6.331317
H	-4.500946	-0.926079	-6.030186
H	-5.822287	-0.401339	-4.975857
H	-0.162228	1.300665	-5.745058
H	0.000367	2.764870	-4.763534
H	0.413334	1.198857	-4.074078
H	2.419245	3.150737	4.150425
H	5.286843	2.285605	1.083502
H	-0.435063	2.770554	2.215636
H	0.184522	3.610073	3.649682
H	-0.091929	4.491359	2.139943
H	5.610862	1.605639	3.648887
H	4.644078	2.550480	4.795980
H	5.796190	3.355225	3.728739
H	4.726091	2.353258	-1.181139
H	3.424535	3.460702	-1.648230
H	3.100256	1.737591	-1.508538
C	1.695317	-2.843141	1.888870
C	1.863056	-0.597369	2.282594
C	2.304873	-3.156829	3.088602
H	1.360794	-3.637939	1.225226
C	2.489142	-0.801610	3.498710
H	1.665212	0.415539	1.945323
H	2.439885	-4.200038	3.345749
H	2.778519	0.065137	4.078494
N	1.465237	-1.587169	1.466800
C	2.735181	-2.119537	3.948446
N	3.349452	-2.376014	5.138889
C	3.747184	-1.280507	6.001759
H	2.885873	-0.681652	6.324566
H	4.230391	-1.684553	6.891635
H	4.462548	-0.615429	5.502676
C	3.545443	-3.745137	5.574805
H	4.058252	-3.743557	6.536850
H	2.591622	-4.274382	5.699707
H	4.163886	-4.308935	4.865302
C	-2.121619	0.983650	6.319056
C	-0.930412	0.434602	6.523423
F	0.886135	0.135693	-1.619428
F	-0.571503	0.357599	0.366765
F	-0.757466	-0.832298	6.829420
F	0.198537	1.107186	6.445917
F	-2.289128	2.251546	6.019628
F	-3.248258	0.313433	6.401524

TS1_A.log

Mg	-0.370681	-1.236833	0.257621
N	1.147066	-2.587499	-0.214928
N	-1.738925	-2.772558	0.622012
C	2.143446	-4.799617	-0.646960
C	0.975757	-3.909811	-0.270443
C	-0.237066	-4.572191	-0.012752
C	-1.487933	-4.058454	0.374293
C	-2.608606	-5.072747	0.484607
C	2.433158	-2.084936	-0.578032
C	3.417004	-1.880921	0.406883
C	4.684204	-1.441239	0.013672
C	4.999422	-1.184996	-1.321938
C	3.989671	-1.347501	-2.273728
C	2.710494	-1.787807	-1.927510
C	3.114683	-2.141067	1.858065
C	6.389037	-0.768328	-1.729740
C	1.647706	-1.951466	-2.980783
C	-3.057209	-2.429462	1.054315
C	-3.411843	-2.536278	2.413320
C	-4.703741	-2.173439	2.803140
C	-5.651580	-1.708477	1.890222
C	-5.275463	-1.616646	0.548819
C	-3.995351	-1.965282	0.112829
C	-2.436704	-3.060376	3.433413
C	-7.027731	-1.294605	2.342583
C	-3.632775	-1.871012	-1.344083
H	2.982790	-4.652475	0.040077
H	2.518180	-4.559053	-1.646859
H	1.857825	-5.852359	-0.626805
H	-0.205720	-5.647426	-0.141044
H	-2.263075	-6.070980	0.211890
H	-3.438495	-4.798341	-0.174845
H	-3.019473	-5.112267	1.497183
H	5.447626	-1.302307	0.777078
H	4.202859	-1.130818	-3.319252
H	2.764839	-3.165007	2.028997
H	2.324770	-1.474150	2.223501
H	4.000172	-1.975693	2.477315
H	6.929739	-0.303909	-0.899638
H	6.980883	-1.631245	-2.060048
H	6.368787	-0.055542	-2.560282
H	2.018379	-1.640827	-3.961405
H	1.302386	-2.988417	-3.062716
H	0.762533	-1.347551	-2.745045
H	-4.973972	-2.258483	3.854503
H	-5.997151	-1.264176	-0.185205
H	-2.739728	-2.777040	4.444891
H	-2.369503	-4.155539	3.408944
H	-1.426494	-2.685031	3.255381
H	-7.361096	-1.882694	3.203307
H	-7.765819	-1.417675	1.544026
H	-7.047028	-0.239861	2.644273
H	-4.500560	-1.589587	-1.944150
H	-3.236960	-2.817688	-1.728298
H	-2.862222	-1.108490	-1.516873
Mg	-0.417737	1.776988	0.157814
N	-1.760732	2.979565	-0.888875
N	0.882470	3.373917	0.513836
C	-2.565092	5.048110	-1.960068
C	-1.542848	4.269379	-1.156105
C	-0.402877	4.990988	-0.761950
C	0.723582	4.582445	-0.024162
C	1.817107	5.619887	0.130439
C	-2.928201	2.390778	-1.463525
C	-4.136085	2.356317	-0.743041
C	-5.272454	1.819842	-1.355301
C	-5.244929	1.309886	-2.654155
C	-4.023552	1.317004	-3.332303
C	-2.862973	1.842624	-2.760835
C	-4.211665	2.902551	0.657313
C	-6.495634	0.791305	-3.315698
C	-1.565347	1.834345	-3.523576
C	2.069389	3.132441	1.272510

C	2.168296	3.540548	2.616825
C	3.336293	3.243559	3.326915
C	4.406683	2.563380	2.748040
C	4.289947	2.183882	1.408480
C	3.143205	2.453246	0.660688
C	1.071481	4.320156	3.294090
C	5.639974	2.228063	3.545571
C	3.067891	2.057149	-0.788311
H	-3.549836	5.008756	-1.484095
H	-2.689390	4.621257	-2.960404
H	-2.269798	6.093256	-2.063418
H	-0.377497	6.020828	-1.097297
H	1.609368	6.500938	-0.478680
H	2.785697	5.206451	-0.167164
H	1.920673	5.939775	1.171291
H	-6.208133	1.809757	-0.799174
H	-3.970455	0.905915	-4.339162
H	-3.880904	3.945510	0.710309
H	-3.570009	2.335621	1.340349
H	-5.233606	2.849670	1.041940
H	-7.243719	0.485721	-2.578072
H	-6.958202	1.560016	-3.947473
H	-6.284747	-0.068774	-3.959324
H	-1.685550	1.344296	-4.493656
H	-1.182874	2.845993	-3.700882
H	-0.784831	1.300520	-2.966542
H	3.405981	3.558385	4.367057
H	5.114913	1.663784	0.926097
H	0.101494	4.147568	2.824923
H	0.999605	4.054187	4.352307
H	1.263375	5.400205	3.250536
H	5.565011	1.229077	3.993149
H	5.794296	2.938500	4.363359
H	6.537197	2.235487	2.918571
H	4.007042	1.610415	-1.120317
H	2.841920	2.914512	-1.432419
H	2.280020	1.312005	-0.962258
C	-1.403833	0.993344	3.557619
C	-0.360349	0.531996	4.575553
F	-0.851945	0.642682	1.945572
F	-0.106328	-0.767507	4.426910
F	-0.807342	0.717877	5.835108
F	0.767335	1.216189	4.437671
F	-1.598923	2.298175	3.591977
F	-2.541518	0.349622	3.679705

TS1_B.log

Mg	-1.320891	-0.642850	-0.650101
N	-3.300576	-0.538641	0.210817
C	-5.768034	-0.599524	0.203454
H	-5.834259	-1.205888	1.109448
H	-6.579013	-0.882878	-0.469644
H	-5.931057	0.439209	0.512780
Mg	1.174490	1.267815	0.209300
N	-2.229975	-0.482632	-2.648697
C	-4.419702	-0.724649	-0.483795
N	-1.020258	-2.811022	-0.968617
C	-4.481396	-0.975311	-1.869368
H	-5.476799	-1.179167	-2.246653
N	-0.491318	-6.921436	-1.653899
C	-3.507862	-0.762939	-2.871129
N	3.176110	0.925491	-0.486980
C	-4.037476	-0.810384	-4.294607
H	-3.432701	-1.468144	-4.925500
H	-3.997191	0.182512	-4.755171
H	-5.072612	-1.155899	-4.317143
N	1.464295	3.404368	-0.080636
C	-3.443077	-0.156006	1.578106
C	-3.614450	-1.118328	2.592608
C	-3.730107	-0.692931	3.920291
H	-3.858610	-1.445128	4.697304
C	-3.688630	0.654662	4.275999
C	-3.519794	1.592106	3.253355
H	-3.492859	2.652511	3.499897
C	-3.399627	1.214348	1.914252
C	-3.716401	-2.585657	2.271941
H	-3.080551	-2.857000	1.427984
C	-3.251292	2.255135	0.840323
H	-4.046572	2.179835	0.090486
C	-1.475905	-0.042737	-3.778652
C	-1.483145	1.334963	-4.090426
C	-0.780654	1.789673	-5.207007
H	-0.805962	2.852406	-5.441963
C	-0.066816	0.922027	-6.037265
C	-0.078257	-0.433562	-5.714947
H	0.453039	-1.135707	-6.356331
C	-0.762412	-0.935522	-4.601436
C	-2.269362	2.294851	-3.241826
H	-3.336117	2.043523	-3.223907
C	-0.733487	-2.416835	-4.336776
H	-1.679369	-2.780439	-3.928573
C	-2.060359	-3.623746	-1.209648
H	-3.040125	-3.150688	-1.228956
C	-1.943315	-4.983210	-1.438154
H	-2.841831	-5.561417	-1.614843
C	-0.663320	-5.584590	-1.434697
C	0.427526	-4.719036	-1.192153
H	1.447988	-5.079028	-1.167404
C	0.197332	-3.373951	-0.967145
H	1.031628	-2.706248	-0.768535
C	-1.643339	-7.778043	-1.853427
H	-2.304826	-7.787993	-0.976575
H	-1.303207	-8.798584	-2.031346
H	-2.230608	-7.464003	-2.725242
C	0.835239	-7.502023	-1.582545
H	1.508527	-7.057893	-2.326065
H	0.768833	-8.570694	-1.788435
H	1.285258	-7.373418	-0.589171
C	2.835678	5.450580	0.017633
H	2.488893	5.933288	-0.902054
H	3.879400	5.728749	0.175575
H	2.232231	5.864317	0.831080
C	2.680507	3.941841	-0.066486
C	3.890619	3.222644	-0.172209
H	4.783850	3.836887	-0.161027
C	4.106101	1.872573	-0.523285
C	5.501421	1.548525	-1.024364
H	5.906664	0.640562	-0.571358
H	6.188029	2.376146	-0.836879
H	5.474355	1.370631	-2.105607
C	0.374862	4.305502	-0.280742
C	0.057279	4.698941	-1.599768
C	-0.997797	5.586459	-1.814850
H	-1.225573	5.888824	-2.836008
C	-1.759336	6.102254	-0.763746
C	-1.423471	5.707612	0.529979
H	-1.985919	6.111305	1.370882
C	-0.374930	4.819142	0.794362
C	0.856510	4.177490	-2.762403
H	0.809501	3.085060	-2.816225
C	-0.067075	4.451708	2.220229
H	1.006591	4.343879	2.394166
C	3.518442	-0.359268	-1.002041
C	4.109895	-1.331656	-0.173443

C	4.412097	-2.587831	-0.710238
H	4.873486	-3.332392	-0.063446
C	4.148791	-2.907851	-2.042445
C	3.557363	-1.927441	-2.843890
H	3.343145	-2.148388	-3.888345
C	3.238413	-0.660281	-2.351100
C	4.445582	-1.020116	1.259037
H	3.589442	-0.590873	1.783864
C	2.613992	0.372631	-3.245909
H	1.609918	0.640714	-2.894385
H	2.516074	0.001583	-4.268369
H	3.198216	1.298996	-3.266700
H	4.760571	-1.920994	1.791594
H	5.259435	-0.288180	1.335077
C	4.523117	-4.251901	-2.612939
H	5.440112	-4.190124	-3.212402
H	4.702020	-4.986935	-1.822057
H	3.738814	-4.643092	-3.270140
H	0.479145	4.581869	-3.705557
H	1.917782	4.439172	-2.684400
H	-0.526928	3.495046	2.490827
H	-0.456901	5.208920	2.907491
C	-2.910729	7.038260	-1.023278
H	-3.142192	7.642801	-0.140965
H	-2.694728	7.720631	-1.851903
H	-3.821352	6.486600	-1.288643
H	-2.166980	3.318827	-3.608723
H	-1.926574	2.271242	-2.201514
H	-0.518284	-2.968332	-5.257387
H	0.043426	-2.674348	-3.609255
C	0.694867	1.438442	-7.230142
H	0.903158	0.639529	-7.948332
H	0.138250	2.223576	-7.752727
H	1.658586	1.870826	-6.933249
H	-3.270475	3.263899	1.259849
H	-2.303035	2.133585	0.300697
H	-3.428746	-3.194476	3.133324
H	-4.742915	-2.868629	2.003592
C	-3.864303	1.089691	5.708439
H	-3.541465	0.311351	6.407137
H	-3.294712	1.999330	5.925564
H	-4.915928	1.309289	5.933014
C	0.530881	1.111659	3.275979
C	0.653343	1.274307	4.643688
C	1.860655	1.771206	5.184696
C	2.876306	2.082553	4.250602
C	2.647828	1.885902	2.900839
N	1.500925	1.402727	2.394965
H	-0.391447	0.721960	2.855248
H	-0.186435	1.004921	5.271163
H	3.837046	2.471286	4.564881
H	3.417785	2.139850	2.176121
N	2.035898	1.941161	6.527656
C	3.305444	2.417685	7.039644
H	3.553694	3.408663	6.639577
H	3.243776	2.502633	8.124894
H	4.128338	1.731403	6.798551
C	0.984847	1.555752	7.449277
H	0.749924	0.485847	7.375247
H	1.311241	1.761034	8.469144
H	0.063922	2.124320	7.269830
C	1.424832	-2.849123	2.898540
C	0.036655	-2.385043	2.477474
F	1.354547	-3.848563	3.816805
F	-0.658612	-3.402341	1.989623
F	0.030291	-1.176151	1.243340
F	-0.604524	-1.844334	3.503898
F	2.106357	-3.328209	1.858660
F	2.111222	-1.855635	3.459813

TS1.log

Mg	0.023251	-1.320164	0.961520
N	1.179064	-2.561549	-0.317520
N	-1.453458	-2.828429	1.164754
C	1.961495	-4.723507	-1.200646
C	1.006633	-3.879069	-0.377668
C	-0.048258	-4.583107	0.239179
C	-1.236268	-4.095237	0.828696
C	-2.334605	-5.124271	1.018762
C	2.157486	-1.985063	-1.181162
C	3.479143	-1.769818	-0.746934
C	4.401521	-1.208238	-1.635824
C	4.052965	-0.834558	-2.933662
C	2.732908	-1.047021	-3.338169
C	1.781065	-1.616607	-2.490369
C	3.911496	-2.140120	0.646127
C	5.057908	-0.205351	-3.863232
C	0.374908	-1.850303	-2.971664
C	-2.762527	-2.480591	1.621058
C	-3.102718	-2.581845	2.984272
C	-4.393661	-2.230447	3.386699
C	-5.354413	-1.770553	2.484326
C	-4.990240	-1.672711	1.140728
C	-3.712969	-2.017247	0.691219
C	-2.106462	-3.072698	3.999929
C	-6.729181	-1.368169	2.951137
C	-3.361841	-1.900600	-0.766299
H	3.004220	-4.510374	-0.947804
H	1.850466	-4.501129	-2.267428
H	1.772687	-5.788266	-1.052935
H	-0.025719	-5.656067	0.087537
H	-1.968900	-6.132457	0.816649
H	-3.162985	-4.917282	0.332046
H	-2.755903	-5.094556	2.026854
H	5.427050	-1.063621	-1.298953
H	2.434255	-0.771722	-4.348447
H	3.604735	-3.155677	0.916097
H	3.464657	-1.471026	1.388730
H	4.998858	-2.074124	0.745232
H	4.953737	0.886626	-3.884457
H	6.083504	-0.429236	-3.554621
H	4.930877	-0.560367	-4.891172
H	0.255335	-1.513138	-4.004738
H	0.096705	-2.909255	-2.926928
H	-0.351041	-1.313189	-2.350908
H	-4.652933	-2.320156	4.440403
H	-5.720629	-1.321221	0.414072
H	-2.556224	-3.111032	4.995844
H	-1.731341	-4.074479	3.761161
H	-1.234666	-2.414621	4.047304
H	-6.747670	-0.320568	3.276303
H	-7.058776	-1.975949	3.799858
H	-7.470144	-1.473954	2.152726
H	-4.228372	-1.587765	-1.353083
H	-2.988098	-2.845513	-1.175716
H	-2.573445	-1.153929	-0.922340
Mg	-0.390380	1.767603	0.592144
N	-1.453542	2.914282	-0.808645
N	0.944154	3.362219	0.958312
C	-2.097679	4.945811	-2.041630
C	-1.233322	4.208635	-1.037325
C	-0.212868	4.971231	-0.438671
C	0.832499	4.571732	0.415032
C	1.909068	5.608817	0.666939

C	-2.403475	2.253288	-1.645381
C	-3.751794	2.140742	-1.260314
C	-4.647736	1.493616	-2.117439
C	-4.246428	0.942788	-3.334539
C	-2.899175	1.054337	-3.687653
C	-1.970580	1.698712	-2.868285
C	-4.232819	2.712683	0.045190
C	-5.226150	0.234223	-4.233713
C	-0.532073	1.820252	-3.293928
C	2.087048	3.081486	1.762575
C	2.115024	3.415158	3.130852
C	3.238035	3.063205	3.890586
C	4.333968	2.403745	3.336641
C	4.288862	2.096265	1.973236
C	3.189975	2.420578	1.177417
C	0.991669	4.182426	3.774476
C	5.531366	2.030393	4.171680
C	3.188759	2.094922	-0.290463
H	-3.160127	4.834160	-1.805267
H	-1.960956	4.537702	-3.048560
H	-1.854049	6.009176	-2.065013
H	-0.175014	6.007683	-0.752286
H	1.679060	6.547376	0.160287
H	2.875999	5.246646	0.300934
H	2.036623	5.811918	1.733401
H	-5.693533	1.425945	-1.822029
H	-2.559850	0.636573	-4.634270
H	-3.906945	3.747974	0.187744
H	-3.843386	2.138567	0.891029
H	-5.324518	2.686398	0.103418
H	-5.128893	-0.855532	-4.152034
H	-6.259049	0.490326	-3.979987
H	-5.064529	0.492520	-5.285594
H	-0.369054	1.332326	-4.258625
H	-0.218859	2.866353	-3.387399
H	0.142135	1.359317	-2.562380
H	3.254727	3.330856	4.946397
H	5.137593	1.595275	1.510675
H	0.027726	3.936801	3.327153
H	0.941637	3.980792	4.847520
H	1.128922	5.265276	3.657174
H	5.611310	0.943034	4.293774
H	5.477441	2.475626	5.169700
H	6.464761	2.367485	3.707334
H	4.141537	1.657477	-0.596803
H	3.002213	2.984190	-0.902328
H	2.402379	1.370500	-0.539009
C	1.410629	-2.851697	3.238811
C	1.570219	-0.598368	3.608054
C	2.106836	-3.147837	4.394722
H	1.052807	-3.653698	2.596351
C	2.273450	-0.785065	4.783381
H	1.346157	0.406838	3.259972
H	2.282246	-4.186523	4.645680
H	2.589154	0.089033	5.337901
N	1.128531	-1.601209	2.832104
C	2.564796	-2.096571	5.224863
N	3.247116	-2.334725	6.380956
C	3.652780	-1.226693	7.224897
H	2.790079	-0.642972	7.571022
H	4.170439	-1.616610	8.101500
H	4.339414	-0.550835	6.700445
C	3.505396	-3.697057	6.805855
H	4.076490	-3.679258	7.734276
H	2.575662	-4.251483	6.989835
H	4.094092	-4.245787	6.060303
C	-2.403915	1.794240	3.573000
C	-1.928494	1.361159	4.953875
F	-1.342077	1.155298	2.316966
F	-1.966178	0.038352	5.073099
F	-2.712921	1.879087	5.929362
F	-0.685265	1.785264	5.183921
F	-2.379676	3.109461	3.425805
F	-3.597280	1.317180	3.289590

TS2.log

Mg	0.264363	-1.388086	-0.092560
N	1.496160	-2.619032	-1.328323
N	-1.214985	-2.874903	0.047293
C	2.276403	-4.780961	-2.215112
C	1.303870	-3.930339	-1.419342
C	0.212349	-4.628292	-0.857586
C	-0.990878	-4.138565	-0.306136
C	-2.102139	-5.162717	-0.173881
C	2.515762	-2.055594	-2.155834
C	3.829763	-1.872584	-1.686175
C	4.786815	-1.322142	-2.547002
C	4.481181	-0.933412	-3.850560
C	3.169331	-1.122711	-4.293155
C	2.185139	-1.679983	-3.476110
C	4.233289	-2.270993	-0.291542
C	5.522179	-0.320267	-4.750591
C	0.793860	-1.902212	-4.003037
C	-2.537708	-2.546714	0.482033
C	-2.910710	-2.710184	1.830058
C	-4.215216	-2.389775	2.213986
C	-5.154266	-1.892493	1.309048
C	-4.755722	-1.727517	-0.018447
C	-3.465932	-2.045594	-0.451246
C	-1.926729	-3.213764	2.852273
C	-6.541308	-1.512299	1.756759
C	-3.085727	-1.874619	-1.897301
H	3.310789	-4.596440	-1.910871
H	2.219835	-4.536971	-3.281246
H	2.057754	-5.843519	-2.096437
H	0.226058	-5.698174	-1.029454
H	-1.743895	-6.164746	-0.414959
H	-2.920028	-4.917039	-0.860147
H	-2.536232	-5.173519	0.829189
H	5.806310	-1.203020	-2.182805
H	2.903840	-0.839245	-5.310373
H	3.820645	-3.242971	-0.006167
H	3.875057	-1.548295	0.448991
H	5.322760	-2.323006	-0.205624
H	5.467612	-0.730453	-5.764572
H	5.387491	0.765091	-4.835540
H	6.533076	-0.496214	-4.371063
H	0.703259	-1.531929	-5.027630
H	0.523863	-2.964697	-4.004990
H	0.054826	-1.389268	-3.381193
H	-4.500549	-2.525295	3.256084
H	-5.467090	-1.339957	-0.745316
H	-2.390962	-3.270853	3.840879
H	-1.540760	-4.209309	2.604964
H	-1.058261	-2.551499	2.922382
H	-6.851900	-2.088035	2.634177
H	-7.278246	-1.679321	0.964869
H	-6.590995	-0.450564	2.027879
H	-3.916839	-1.459336	-2.471689
H	-2.793423	-2.827112	-2.354946
H	-2.233407	-1.197631	-2.014602
Mg	-0.637284	1.824075	-0.518333
N	-1.576865	2.937950	-2.023097
N	0.859112	3.250621	-0.213472

C	-2.005550	4.964731	-3.345656
C	-1.238181	4.195693	-2.289160
C	-0.172580	4.892958	-1.683096
C	0.825522	4.449737	-0.797361
C	1.956093	5.428149	-0.548000
C	-2.583224	2.327038	-2.834724
C	-3.951165	2.433874	-2.527874
C	-4.877761	1.757118	-3.333264
C	-4.495251	1.000751	-4.437215
C	-3.130248	0.934261	-4.739536
C	-2.170763	1.580940	-3.963489
C	-4.460551	3.296470	-1.403004
C	-5.510418	0.288823	-5.293618
C	-0.715817	1.520780	-4.342028
C	2.012782	2.911735	0.554242
C	2.136507	3.270084	1.910482
C	3.277648	2.862376	2.615992
C	4.301541	2.130003	2.019303
C	4.164174	1.801009	0.666194
C	3.044448	2.176907	-0.075743
C	1.115272	4.131396	2.606239
C	5.521617	1.705917	2.795165
C	2.956906	1.850129	-1.541740
H	-3.056660	5.074306	-3.061619
H	-1.993695	4.430945	-4.301266
H	-1.582808	5.959166	-3.495971
H	-0.051089	5.913858	-2.024572
H	1.817835	6.344310	-1.123774
H	2.917534	4.984577	-0.827378
H	2.028880	5.693570	0.510457
H	-5.935595	1.842616	-3.088195
H	-2.803058	0.366622	-5.609349
H	-3.651158	3.678263	-0.780544
H	-5.145595	2.740136	-0.757257
H	-5.016124	4.155791	-1.800077
H	-5.465895	0.630309	-6.334451
H	-5.338189	-0.793775	-5.305185
H	-6.528225	0.459464	-4.931359
H	-0.577253	0.930604	-5.251943
H	-0.306139	2.521218	-4.523595
H	-0.115348	1.072219	-3.544985
H	3.369375	3.150871	3.662606
H	4.957312	1.247501	0.166649
H	0.153022	4.123918	2.093703
H	0.961028	3.803771	3.638644
H	1.453888	5.174629	2.653992
H	5.542817	0.619293	2.944676
H	5.553398	2.179975	3.780855
H	6.443969	1.972887	2.267601
H	3.848832	1.317091	-1.877337
H	2.848921	2.758551	-2.145265
H	2.090811	1.220428	-1.769187
C	1.677024	-2.982854	2.072132
C	1.942022	-0.757038	2.531075
C	2.362716	-3.358441	3.211212
H	1.282593	-3.743530	1.401371
C	2.646681	-1.025763	3.689822
H	1.759839	0.271538	2.237954
H	2.488160	-4.413833	3.418309
H	3.006103	-0.190763	4.277261
N	1.449406	-1.705735	1.716213
C	2.878311	-2.365827	4.077939
N	3.557219	-2.682231	5.216658
C	4.045430	-1.629595	6.087281
H	3.226310	-1.007004	6.469345
H	4.553143	-2.078992	6.940861
H	4.762947	-0.979098	5.571736
C	3.762713	-4.071738	5.578510
H	4.332721	-4.118503	6.506528
H	2.811535	-4.595754	5.738593
H	4.329246	-4.609480	4.807939
C	-2.224894	2.109691	1.340013
C	-1.581895	1.194928	2.234905
F	-0.066932	0.124775	-1.273425
F	-0.405357	0.382093	1.126702
F	-2.154424	0.099929	2.625489
F	-0.701231	1.630607	3.087276
F	-2.214947	3.398110	1.809354
F	-3.518890	1.742389	1.108083

6. References

(1) Sheldrick, G. Crystal Structure Refinement with SHELXL. *Acta Crystallogr. Sect. C* **2015**, *71*, 3–8.

(2) Dolomanov, O. V; Bourhis, L. J.; Gildea, R. J.; Howard, J. A. K.; Puschmann, H. OLEX2: A Complete Structure Solution, Refinement and Analysis Program. *J. Appl. Crystallogr.* **2009**, *42*, 339–341.

(3) Hicks, J.; Juckel, M.; Paparo, A.; Dange, D.; Jones, C. Multigram Syntheses of Magnesium(I) Compounds Using Alkali Metal Halide Supported Alkali Metals as Dispersible Reducing Agents. *Organometallics* **2018**, *37*, 4810–4813.

(4) Bakewell, C.; Ward, B. J.; White, A. J. P.; Crimmin, M. R. A Combined Experimental and Computational Study on the Reaction of Fluoroarenes with Mg–Mg, Mg–Zn, Mg–Al and Al–Zn Bonds. *Chem. Sci.* **2018**, *9*, 2348–2356.

(5) Fagnani, D. E.; Kim, D.; Camarero, S. I.; Alfaro, J. F.; McNeil, A. J. Using Waste Poly(Vinyl Chloride) to Synthesize Chloroarenes by Plasticizer-Mediated Electro(de)Chlorination. *Nat. Chem.* **2023**, *15*, 222-229.

(6) Bakewell, C.; White, A. J. P.; Crimmin, M. R. Addition of Carbon–Fluorine Bonds to a Mg(I)–Mg(I) Bond: An Equivalent of Grignard Formation in Solution. *J. Am. Chem. Soc.* **2016**, *138*, 12763–12766.

(7) Bonyhady, S. J.; Jones, C.; Nembenna, S.; Stasch, A.; Edwards, A. J.; McIntyre, G. J. β-Diketiminate-Stabilized Magnesium(I) Dimers and Magnesium(II) Hydride Complexes: Synthesis, Characterization, Adduct Formation, and Reactivity Studies.

Chem. – A Eur. J. **2010**, *16*, 938–955.
(8) Sati, G. C.; Martin, J. L.; Xu, Y.; Malakar, T.; Zimmerman, P. M.; Montgomery, J. Fluoride Migration Catalysis Enables Simple, Stereoselective, and Iterative Glycosylation. *J. Am. Chem. Soc.* **2020**, *142*, 7235–7242.
(9) Bläsing, K.; Ellinger, S.; Harloff, J.; Schulz, A.; Sievert, K.; Täschler, C.; Villinger, A.; Zur Täschler, C. Lewis Acid Catalyzed Synthesis of Cyanidophosphates. *Chem. – A Eur. J.* **2016**, *22*, 4175–4188.
(10) Sheldon, D. J.; Crimmin, M. R. Complete Deconstruction of SF6 by an Aluminium(i) Compound. *Chem. Commun.* **2021**, *57*, 7096–7099.
(11) Frisch, M. J.; Trucks, G. W.; Schlegel, H. B.; Scuseria, G. E.; Robb, M. A.; Cheeseman, J. R.; Scalmani, G.; Barone, V.; Mennucci, B.; Petersson, G. A. Gaussian 09; Revision D.01, Gaussian 09; Revision D.01, Gaussian Inc., 2009.
(12) Perdew, J. P.; Burke, K.; Wang, Y. Generalized Gradient Approximation for the Exchange-Correlation Hole of a Many-Electron System. *Phys. Rev. B* **1996**, *54*, 16533–16539.
(13) Becke, A. D. Density-functional Thermochemistry. III. The Role of Exact Exchange. *J. Chem. Phys.* **1993**, *98*, 5648–5652.
(14) Perdew, J. P.; Burke, K.; Ernzerhof, M. Generalized Gradient Approximation Made Simple. *Phys. Rev. Lett.* **1996**, *77*, 3865–3868.
(15) Perdew, J. P.; Chevary, J. A.; Vosko, S. H.; Jackson, K. A.; Pederson, M. R.; Singh, D. J.; Fiolhais, C. Erratum: Atoms, Molecules, Solids, and Surfaces: Applications of the Generalized Gradient Approximation for Exchange and Correlation. *Phys. Rev. B* **1993**, *48*, 4978.
(16) Perdew, J. P.; Chevary, J. A.; Vosko, S. H.; Jackson, K. A.; Pederson, M. R.; Singh, D. J.; Fiolhais, C. Atoms, Molecules, Solids, and Surfaces: Applications of the Generalized Gradient Approximation for Exchange and Correlation. *Phys. Rev. B* **1992**, *46*, 6671–6687.
(17) Zhao, Y.; Truhlar, D. G. The M06 Suite of Density Functionals for Main Group Thermochemistry, Thermochemical Kinetics, Noncovalent Interactions, Excited States, and Transition Elements: Two New Functionals and Systematic Testing of Four M06-Class Functionals and 12 Other Function. *Theor. Chem. Acc.* **2008**, *120*, 215–241.
(18) Lledós, A. Computational Organometallic Catalysis: Where We Are, Where We Are Going. *Eur. J. Inorg. Chem.* **2021**, *2021*, 2547–2555.
(19) Weigend, F.; Ahlrichs, R. Balanced Basis Sets of Split Valence, Triple Zeta Valence and Quadruple Zeta Valence Quality for H to Rn: Design and Assessment of Accuracy. *Phys. Chem. Chem. Phys.* **2005**, *7*, 3297–3305.
(20) Tomasi, J.; Mennucci, B.; Cammi, R. Quantum Mechanical Continuum Solvation Models. *Chem. Rev.* **2005**, *105*, 2999–3094.
(21) Grimme, S.; Antony, J.; Ehrlich, S.; Krieg, H. A Consistent and Accurate Ab Initio Parametrization of Density Functional Dispersion Correction (DFT-D) for the 94 Elements H-Pu. *J. Chem. Phys.* **2010**, *132*, 154104.
(22) Grimme, S.; Ehrlich, S.; Goerigk, L. Effect of the Damping Function in Dispersion Corrected Density Functional Theory. *J. Comput. Chem.* **2011**, *32*, 1456–1465.
(23) Fukui, K. The Path of Chemical Reactions - the IRC Approach. *Acc. Chem. Res.* **1981**, *14*, 363–368.
(24) Dykstra, C. E. *Theory and Applications of Computational Chemistry : The First Forty Years*; Elsevier, 2005.
(25) Dennington, R.; Keith, T.; Milliam, J. GaussView 5.0, GaussView 5.0, Semichem Inc., Shawnee Mission, KS, 2009.
(26) Glendening, E. D.; Badenhoop, J. K.; Reed, A. E.; Carpenter, J. E.; Bohmann, J. A.; Morales, C. M.; Landis, C. R.; Weinhold, F. NBO 6.0, NBO 6.0, Theoretical Chemistry Institute, University of Wisconsin, Madison, 2013.
(27) Yuvaraj, K.; Douair, I.; Jones, D. D. L.; Maron, L.; Jones, C. Sterically Controlled Reductive Oligomerisations of CO by Activated Magnesium(i) Compounds: Deltate vs. Ethenediolate Formation. *Chem. Sci.* **2020**, *11*, 3516–3522.

(28) Chai, J.-D.; Head-Gordon, M. Systematic Optimization of Long-Range Corrected Hybrid Density Functionals. *J. Chem. Phys.* **2008**, *128*, 84106.
(29) Chai, J.-D.; Head-Gordon, M. Long-Range Corrected Hybrid Density Functionals with Damped Atom–Atom Dispersion Corrections. *Phys. Chem. Chem. Phys.* **2008**, *10*, 6615–6620.
(30) Coates, G.; Ward, B. J.; Bakewell, C.; White, A. J. P.; Crimmin, M. R. Reactions of Fluoroalkanes with Mg−Mg Bonds: Scope, Sp 3 C−F/Sp 2 C−F Coupling and Mechanism. *Chem. - A Eur. J.* **2018**, *24*, 16282–16286.
